# Regulated Cell Death in Reproductive Biology: Mechanisms and Therapeutic Opportunities

**DOI:** 10.3390/cells15201837

**Published:** 2026-10-10

**Authors:** Sofoklis Stavros, Efthalia Moustakli, Nikolaos Kathopoulis, Stefanos Dafopoulos, Dimos Sioutis, Athanasios Zikopoulos, Ismini Anagnostaki, Anastasios Potiris, Konstantinos Dafopoulos, Peter Drakakis

**Affiliations:** 1 Third Department of Obstetrics and Gynecology, University General Hospital “ATTIKON”, Medical School, National and Kapodistrian University of Athens, 12462 Athens, Greece; nickatho@gmail.com (N.K.); dsioutis@gmail.com (D.S.); apotiris@med.uoa.gr (A.P.); pdrakakis@hotmail.com (P.D.); 2 Department of Nursing, School of Health Sciences, University of Ioannina, 4th Kilometer National Highway Str. Ioannina-Athens, 45500 Ioannina, Greece; 3 University General Hospital of Patras, 26504 Rio, Greece; stefanosntf2001@gmail.com; 4 Department of Reproductive Medicine and Surgery, University College London Hospitals NHS Foundation Trust, 235 Euston Road, London NW1 2BU, UK; thanzik92@gmail.com; 5 Medical School, National and Kapodistrian University of Athens, 11528 Athens, Greece; isanagnostaki3@gmail.com; 6 Department of Obstetrics and Gynaecology, Faculty of Medicine, School of Health Sciences, University of Thessaly, 41110 Larissa, Greece; kdafop@uth.gr

**Keywords:** ferroptosis, pyroptosis, necroptosis, cuproptosis, PANoptosis, oxidative stress, mitochondrial dysfunction, infertility, embryo implantation, placental development, biomarkers, precision medicine

## Abstract

**Highlights:**

**What are the main findings?**

- Regulated cell death is integral to reproductive physiology and pathology, with apoptosis remaining the best-established pathway and ferroptosis emerging as the most extensively investigated non-apoptotic RCD mechanism across reproductive disorders.
- The contribution of RCD is highly context-, cell type-, and stage-dependent, and molecular or transcriptomic signatures alone are insufficient to demonstrate execution of a specific cell-death pathway.

**What are the implications of the main findings?**

- Future studies should prioritize functional validation of RCD mechanisms using pathway-specific inhibition, rescue, or genetic approaches together with human-based and physiologically relevant experimental models.
- Translation of RCD-targeted strategies into reproductive medicine will require pathway specificity, reproductive safety, clinically relevant outcomes, and functionally validated RCD phenotypes that may ultimately support biologically informed patient stratification.

**Abstract:**

Regulated cell death (RCD) has emerged as a fundamental determinant of cellular homeostasis and tissue remodeling, extending far beyond the classical concept of apoptosis. The identification of emerging RCD pathways, including ferroptosis, pyroptosis, necroptosis, cuproptosis, PANoptosis, and autophagy-dependent cell death, has expanded our understanding of reproductive physiology and disease. RCD mechanisms contribute to reproductive homeostasis and have been increasingly implicated in gametogenesis, folliculogenesis, embryonic development, implantation, placentation, and reproductive aging, while their dysregulation has been associated with infertility, recurrent implantation failure, recurrent pregnancy loss, pregnancy complications, endometriosis, polycystic ovary syndrome (PCOS), gynecological malignancies, and male reproductive disorders. This review provides a comprehensive overview of the molecular mechanisms governing RCD across reproductive physiology and disease, emphasizing the complex interplay between oxidative stress (OS), mitochondrial dysfunction, inflammation, immune regulation, metabolic reprogramming, and genetic and epigenetic networks. Particular attention is given to the contribution of emerging cell death pathways to reproductive disorders and their interactions with environmental and lifestyle factors, including endocrine-disrupting chemicals. In addition, current evidence regarding RCD-associated biomarkers, multi-omics approaches, and therapeutic strategies targeting specific cell death pathways is critically evaluated, with particular emphasis on distinguishing associative molecular signatures from functional evidence of RCD execution. By integrating recent advances from basic, translational, and clinical research, this review highlights the context-dependent role of RCD in reproductive biology and disease and identifies current limitations and future opportunities for RCD-informed diagnostics, patient stratification, and targeted therapeutic interventions in reproductive medicine.

## 1. Introduction

Regulated cell death (RCD) is characterized by genetically and biochemically regulated mechanisms that eliminate cells in response to stimuli such as developmental signals, cellular stresses, or irreversible damage [1]. Although apoptosis has historically been the most extensively studied form of RCD in reproductive science, recent research has expanded to other mechanisms, including ferroptosis, pyroptosis, necroptosis, autophagy-dependent cell death, and, more recently, cuproptosis and PANoptosis [2,3,4,5]. These RCD modalities differ in their initiating stimuli, molecular mechanisms, metabolic dependencies, and effects on surrounding cells. Notably, these cell death pathways do not operate independently but exhibit substantial molecular cross-talk involving mitochondrial dysfunction, redox imbalance, inflammation, metabolic stress, and immune signaling. This interplay can influence the predominant cell death phenotype depending on the cell type and specific stimulus [1,6,7].

Reproductive biology provides a particularly relevant context for studying RCD, as it contributes to essential physiological processes throughout the reproductive lifespan. During spermatogenesis, RCD helps maintain germ cell homeostasis and eliminate defective cells [8,9,10], whereas in the ovary it plays a key role in follicular selection and atresia [11,12]. Furthermore, mechanisms of cell death contribute to oocyte competency, embryogenesis, cyclic remodelling of the endometrium, implantation, trophoblast function and placentation [13]. Thus, in these contexts, RCD should not be regarded solely as a pathological process. Rather, reproductive function requires precise spatiotemporal regulation of cell survival and death, whereas dysregulation of RCD may contribute to tissue dysfunction and infertility.

This biological context is particularly relevant because reproductive cells are exposed to multiple factors that influence cell-death signaling. Redox homeostasis, mitochondrial function, lipid metabolism, metal homeostasis, inflammatory signaling, and immune-cell interactions are closely integrated with reproductive physiology and can modulate susceptibility to multiple RCD pathways [14,15,16]. For example, ferroptosis is characterized by iron-dependent lipid peroxidation, pyroptosis by gasdermin-mediated membrane pore formation and inflammatory signaling, and necroptosis by receptor-interacting protein kinase (RIPK)-dependent signaling [17,18,19]. Cuproptosis results from copper-dependent targeting of lipoylated mitochondrial proteins and consequent proteotoxic stress, whereas PANoptosis involves integrated regulation of pyroptotic, apoptotic, and necroptotic machinery within a coordinated inflammatory cell-death program [20,21,22].

An increasing number of experimental and clinical studies indicate that dysregulated RCD signaling may contribute to reproductive disease. Several RCD pathways, including ferroptosis, pyroptosis, and necroptosis, have been implicated in conditions including ovarian dysfunction, polycystic ovary syndrome (PCOS), endometriosis, male infertility, recurrent implantation failure, recurrent pregnancy loss, and placental disorders such as preeclampsia and fetal growth restriction [23,24]. Related biological pathways are also being investigated in gynecological cancers, where both resistance to apoptosis and susceptibility to alternative RCD modalities may influence disease progression and therapeutic response [25]. Nevertheless, the level of evidence varies across reproductive conditions and RCD modalities. In some areas, experimental evidence is derived primarily from cellular and animal models, while human data are limited to molecular expression profiles, biomarker analyses, or computational signatures. Specifically, the identification of an “RCD-related” gene signature does not necessarily indicate the activation or execution of a specific cell death pathway [25]. Therefore, it is crucial to distinguish between demonstrated cell death processes and molecular correlations when assessing the translational implications of this rapidly developing field.

RCD has implications not only for disease processes but also for biomarker discovery. Molecular components of cell death pathways are increasingly being investigated as biomarkers of reproductive disorders, while multi-omics approaches provide a more comprehensive characterization of RCD-associated molecular profiles [6,7,26]. In parallel, pharmacological modulation of ferroptosis, pyroptosis, necroptosis, autophagy, and other RCD-related pathways has attracted increasing translational interest [27]. However, most RCD-related approaches in reproductive medicine remain preclinical, and the involvement of an RCD pathway in disease pathogenesis should not be considered evidence of its therapeutic relevance. It is therefore essential to establish the level of experimental and clinical validation before integrating RCD-related biomarkers and interventions into precision reproductive medicine.

Accordingly, this review highlights the biological importance of RCD mechanisms throughout reproductive biology, concentrating specifically on apoptosis, ferroptosis, pyroptosis, necroptosis, cuproptosis, autophagy-dependent cell death, and PANoptosis. The molecular basis for each RCD modality and the interactions among these pathways are first described. Their physiological roles in gametogenesis, ovarian biology, embryogenesis, implantation, placentation, and reproductive aging are then examined. The review subsequently evaluates primary evidence linking RCD mechanisms to reproductive disorders, including infertility, implantation failure, pregnancy loss, placental disorders, and gynecologic cancers, with particular emphasis on oxidative stress (OS), mitochondrial dysfunction, inflammation, immune signaling, metabolism, and environmental factors. Finally, the current evidence on RCD-related biomarkers, multi-omics profiling, and therapeutic targeting is critically assessed, distinguishing among mechanistic, preclinical, and human evidence. Through this approach, the review aims to distinguish the mechanistic roles of RCD in reproductive biology from disease-associated findings and to identify areas with potential for future clinical translation.

## 2. Literature Search Strategy

This narrative review was informed by a structured search of the literature on RCD in reproductive physiology and disease. Electronic literature searches were conducted using PubMed/MEDLINE, Scopus, and ScienceDirect for publications available up to 15 September 2026. The search covered apoptosis, ferroptosis, pyroptosis, necroptosis, autophagy-dependent cell death, cuproptosis, and PANoptosis in reproductive contexts. The general search structure combined RCD-related terms (“apoptosis” OR “ferroptosis” OR “pyroptosis” OR “necroptosis” OR “autophagy-dependent cell death” OR “cuproptosis” OR “PANoptosis”) with reproductive terms (“female infertility” OR “male infertility” OR “polycystic ovary syndrome” OR “endometriosis” OR “premature ovarian insufficiency” OR “diminished ovarian reserve” OR “implantation failure” OR “recurrent pregnancy loss” OR “preeclampsia” OR “fetal growth restriction” OR “gestational diabetes” OR “placenta” OR “ovary” OR “testis” OR “sperm” OR “endometrium” OR “trophoblast” OR “ovarian cancer” OR “endometrial cancer” OR “cervical cancer” OR “gynecological malignancy” OR “reproductive toxicity” OR “endocrine-disrupting chemicals” OR “environmental pollutants”). Searches were adapted to the syntax and functionality of each database. Reference lists of relevant primary studies and review articles were also examined to identify additional pertinent publications.

Studies were considered for inclusion when they investigated an RCD pathway in relation to reproductive physiology, reproductive or pregnancy-related disorders, gynecological malignancies, or reproductive toxicology and provided molecular, experimental, clinical, or therapeutic evidence relevant to the scope of the review. Primary experimental and clinical studies were prioritized when evaluating the involvement of specific RCD pathways and their therapeutic modulation, while reviews and other secondary sources were used to provide biological context and identify additional relevant literature. Studies not directly relevant to reproductive biology or lacking information pertinent to RCD mechanisms were excluded. As this article was designed as a narrative rather than a systematic review, no formal PRISMA-based study-selection process or risk-of-bias assessment was undertaken.

Evidence was interpreted according to the level of mechanistic support for the proposed RCD pathway. Changes in pathway-associated genes, proteins, or computational signatures alone were considered associative and were not regarded as evidence of RCD execution. Greater mechanistic weight was assigned to studies demonstrating pathway-specific biochemical or morphological features together with functional inhibition or rescue, genetic manipulation, or other experiments establishing pathway dependency. Similarly, inflammasome activation was distinguished from gasdermin-mediated pyroptosis, autophagic activity from autophagy-dependent cell death, and RCD-related transcriptional signatures from functional evidence of activation of the corresponding death program. This framework was applied consistently when evaluating studies included in Table 1 and Table 2. Evidence was considered across five complementary levels: (i) molecular association, including changes in gene or protein expression, biomarkers, or computational signatures; (ii) biochemical or morphological evidence of pathway execution; (iii) functional evidence based on pharmacological inhibition or rescue; (iv) genetic evidence demonstrating dependency on specific pathway components; and (v) validation in primary human cells or patient-derived tissues [1]. The strongest mechanistic evidence was assigned to studies demonstrating pathway dependency through pharmacological inhibition or rescue, genetic manipulation, or complementary functional approaches. Human evidence was additionally distinguished from preclinical mechanistic evidence when assessing translational relevance, to avoid extrapolating experimental pathway validation directly to clinical disease.

## 3. Molecular Mechanisms of RCD

RCD involves specific molecular mechanisms characterized by distinct initiating stimuli, signaling events, modes of execution, and effects on surrounding cells and tissues [6]. However, these RCD modalities are interconnected through shared molecular and metabolic mechanisms involving mitochondrial function, redox homeostasis, inflammatory signaling, nutrient availability, and metal homeostasis. Such interplay is especially important for reproductive tissues, whose functional states vary dynamically during development, tissue remodeling, and adaptation to physiological stress. Therefore, identification of the molecular machinery underlying a particular RCD mechanism is critical before discussing its physiological and pathophysiological functions in reproduction [1,7,14].

**Figure 1:**
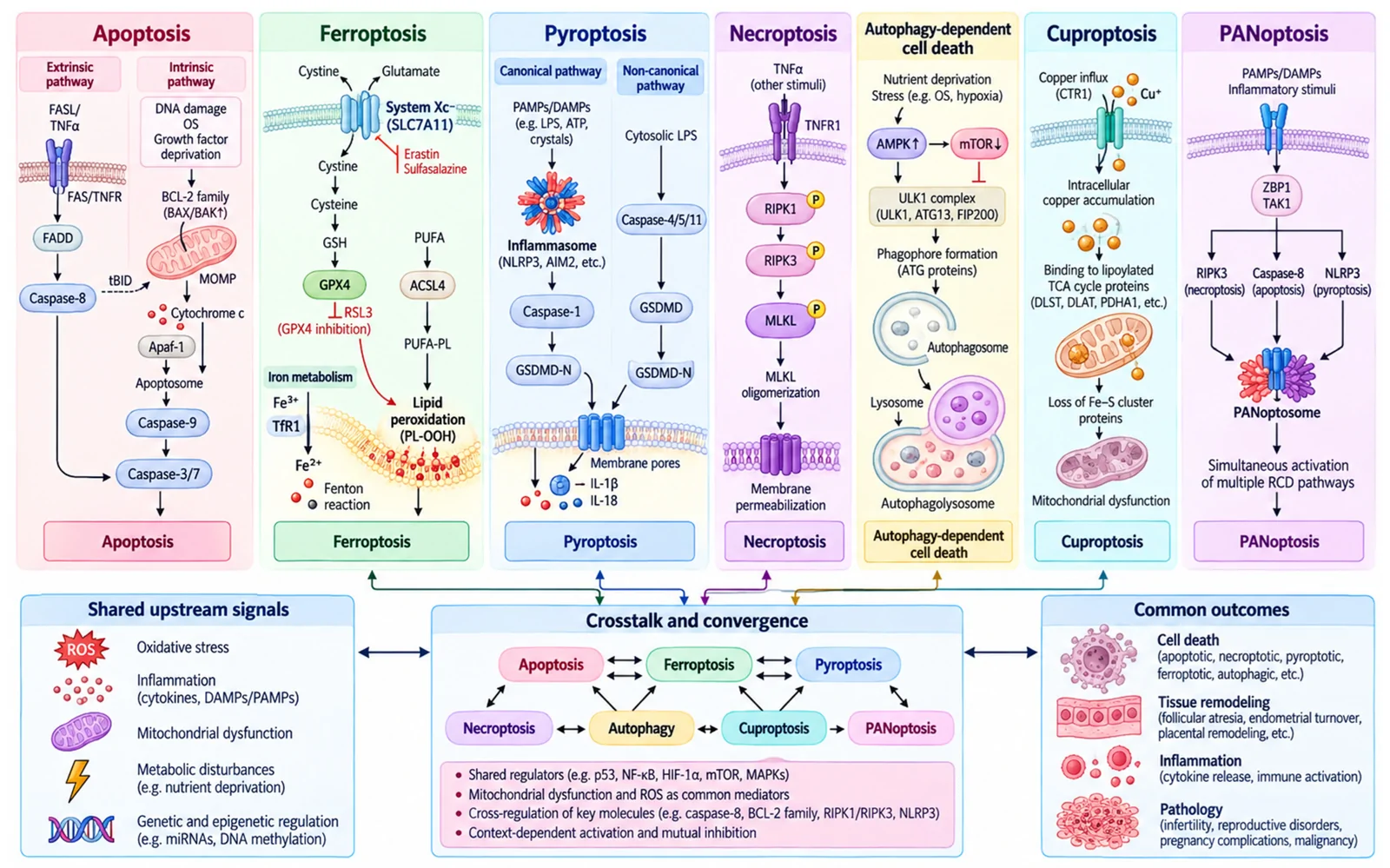
Molecular mechanisms of major regulated cell death pathways and their interconnections. Schematic representation of the key signaling mechanisms underlying apoptosis, ferroptosis, pyroptosis, necroptosis, autophagy-dependent cell death, cuproptosis, and PANoptosis, highlighting major molecular regulators, shared upstream signals, pathway crosstalk, and common cellular outcomes. Abbreviations are defined in the main text. This figure is an original illustration created by the authors using ChatGPT (GPT-5.6 Sol, OpenAI) and was not reproduced or adapted from previously published material.

The major RCD modalities are governed by distinct molecular machinery but exhibit substantial interconnection through shared upstream signals and crosstalk involving oxidative stress, mitochondrial dysfunction, metabolic perturbation, and inflammatory signaling (Figure 1).

### 3.1. Apoptosis

Apoptosis is a caspase-dependent form of RCD characterized by distinct morphological and biochemical features. Morphologically, apoptosis is characterized by cell shrinkage, chromatin condensation, DNA fragmentation, and formation of membrane-enclosed apoptotic bodies, while plasma membrane integrity is preserved during the execution phase [28]. Apoptosis is primarily regulated through the intrinsic and extrinsic pathways.

The intrinsic pathway is regulated mainly by the B-cell lymphoma 2 (BCL-2) family proteins and is induced by intracellular signals including DNA damage, OS, growth factor deprivation, and mitochondrial dysfunction. Pro-apoptotic BAX and BAK are responsible for mitochondrial outer membrane permeabilization, which leads to cytochrome c release and apoptosome assembly via APAF1 (apoptotic protease activating factor 1). The resulting apoptosome recruits caspase-9, which then activates executioner caspases-3 and -7 [29,30]. The extrinsic pathway is activated by stimulation of cell-surface death receptors, such as FAS and tumor necrosis factor receptors, leading to assembly of the death-inducing signaling complex and activation of caspase-8. Cross-talk between these pathways is mediated by molecules such as BID [31,32,33].

While apoptosis remains essential for physiological cell turnover, its molecular pathways exhibit complex cross-talk with other forms of RCD. For example, caspase-8 promotes extrinsic apoptosis while suppressing necroptosis, indicating that cell fate depends on the balance between competing death pathways [34,35].

### 3.2. Ferroptosis

Ferroptosis is an iron-dependent form of RCD driven by uncontrolled phospholipid peroxidation rather than caspase activation [4]. It occurs when the balance between lipid peroxidation and cellular antioxidant defenses is disrupted. This dependence on iron, lipid peroxidation, and redox imbalance distinguishes ferroptosis from other forms of cell death, including apoptosis and necrosis [36,37].

A key protective mechanism involves the cystine/glutamate antiporter system Xc^−^, which consists of SLC7A11 and SLC3A2. Cystine uptake supports glutathione synthesis, which provides the reducing capacity required by GPX4 to convert lipid hydroperoxides into less reactive lipid alcohols. Inhibition of system Xc^−^, depletion of glutathione, or inhibition of GPX4 can result in the accumulation of lipid hydroperoxides and eventually ferroptotic cell death [18,38,39]. Additional ferroptosis defense mechanisms include the FSP1–coenzyme Q10 pathway and mitochondrial DHODH-dependent antioxidant activity [40].

Ferroptosis is also influenced by the control of membrane lipids and iron metabolism. An increase in the labile iron pool promotes Fenton chemistry, while enzymes such as acyl-CoA synthetase long-chain family member 4 (ACSL4) and lysophosphatidylcholine acyltransferase 3 (LPCAT3) facilitate the incorporation of polyunsaturated fatty acids into membrane phospholipids, increasing their susceptibility to oxidation [41,42]. Cellular iron availability is further regulated by iron uptake, storage, and export mechanisms, particularly transferrin receptor-mediated iron uptake, ferritin storage, and ferroportin-mediated iron export, which collectively influence the labile iron pool and susceptibility to ferroptosis. Therefore, increased lipid peroxidation or altered expression of GPX4, SLC7A11, ACSL4, or iron-regulatory proteins may indicate perturbation of ferroptosis-related pathways but does not confirm ferroptotic cell death. Stronger evidence requires demonstration that cell death is dependent on ferroptosis-related mechanisms, ideally through functional inhibition, rescue, or genetic manipulation [43].

### 3.3. Pyroptosis

Pyroptosis is a lytic and highly pro-inflammatory form of RCD executed by members of the gasdermin protein family. In the canonical pathway, recognition of pathogen-associated or damage-associated molecular patterns promotes the assembly of inflammasome complexes, such as the NLRP3 and AIM2 inflammasomes. Inflammasome assembly leads to the activation of caspase-1, which cleaves gasdermin D (GSDMD) and processes the pro-inflammatory cytokines pro-IL-1β and pro-IL-18 into their mature forms [19,44,45].

The released N-terminal fragment of GSDMD oligomerizes in the plasma membrane to create pores, leading to ionic imbalance, membrane lysis, and release of pro-inflammatory molecules. The non-canonical pathway is activated by intracellular lipopolysaccharide through caspases-4 and -5 in humans and caspase-11 in mice, leading to GSDMD cleavage and secondary activation of NLRP3 [44,46,47]. Other gasdermin family members also contribute to cross-talk between RCD pathways; for example, caspase-3-mediated cleavage of gasdermin E (GSDME) can shift apoptosis toward pyroptosis in cells with sufficient GSDME expression [48].

Therefore, increased inflammasome activity and elevated IL-1β and IL-18 levels do not necessarily indicate the occurrence of pyroptotic cell death. Evidence of gasdermin activation and membrane pore formation provides more specific support for pyroptosis than inflammatory markers alone.

### 3.4. Necroptosis

Necroptosis is a regulated lytic form of cell death that can be triggered by death receptors and innate immune signaling pathways, particularly when caspase-8-dependent apoptosis is inhibited or ineffective. The core components of the necroptotic machinery are receptor-interacting protein kinase 1 (RIPK1), RIPK3, and mixed lineage kinase domain-like protein (MLKL) [49,50,51].

Under appropriate signaling conditions, the RIP homotypic interaction motifs of RIPK1 and RIPK3 interact to form a signaling complex known as the necrosome. The phosphorylation of MLKL by RIPK3 leads to its oligomerization and membrane translocation. In its active state, MLKL induces membrane permeabilization, causing an ionic imbalance, cell swelling, membrane rupture, and the release of DAMPs [52,53]. Due to its ability to induce membrane lysis, necroptosis may contribute to local inflammation.

The balance between necroptosis and apoptosis is tightly regulated. Caspase-8 suppression permits RIPK3–MLKL signaling downstream of death receptors by preventing cleavage of key pathway components [54,55,56]. The detection of phosphorylated RIPK3 and MLK, in conjunction with functional rescue following pathway inhibition, provides stronger evidence for necroptosis than changes in RIPK1 expression alone.

### 3.5. Cuproptosis

Cuproptosis is a recently characterized form of RCD that is dependent on copper and closely linked to mitochondrial metabolism. Mechanistically, copper binds directly to lipoylated components of the tricarboxylic acid cycle, promoting their aggregation and inducing proteotoxic stress [57,58].

One of the main regulators of this process is ferredoxin 1 (FDX1), which participates in copper-mediated cytotoxicity and protein lipoylation. Excess intracellular copper promotes the aggregation of mitochondrial lipoylated proteins, particularly components of the pyruvate dehydrogenase complex, while also disrupting iron–sulfur proteins and inducing proteotoxic stress [59,60,61]. Thus, cells that rely on mitochondrial respiration are more vulnerable to cuproptosis than those that rely on glycolysis.

Given the relatively recent characterization of cuproptosis, caution is warranted when interpreting studies in reproductive biology. Signatures composed of genes implicated in the machinery of cuproptosis or in copper-associated metabolic processes may demonstrate biological links or putative molecular phenotypes, but do not establish that cuproptotic cell death is occurring in the tissue under investigation.

### 3.6. Autophagy-Dependent Cell Death

Autophagy primarily functions as an adaptive cellular quality-control mechanism rather than as a cell death process. Autophagy involves the sequestration of damaged organelles, protein complexes, and other intracellular components into autophagosomes, followed by their lysosomal degradation, thereby promoting cell survival under nutrient deprivation or cellular stress [62,63]. This process involves initiation by the ULK1 complex, formation of the phagophore by the class III phosphatidylinositol 3-kinase complex that includes BECN1, and the activity of ATG proteins required for autophagosome elongation and LC3 lipidation [64].

The term “autophagy-dependent cell death” should be used more rigorously than the broader and often imprecise designation “autophagic cell death.” Mechanistically, autophagy-dependent cell death is defined as cell death that requires the autophagic machinery, such that genetic disruption of essential autophagy components prevents or substantially reduces cell death; pharmacological inhibition may provide additional supportive evidence but requires careful interpretation because of potential off-target effects [63,65]. Elevated LC3-II, reduced p62/SQSTM1, or increased autophagosome abundance may indicate altered autophagic activity or flux but do not necessarily demonstrate autophagy-dependent cell death. This is especially true for reproductive tissues where autophagy often serves as an adaptation mechanism under metabolic or OS [66,67].

### 3.7. Crosstalk Between RCD Pathways and PANoptosis

Increasing evidence suggests that multiple RCD pathways can be activated simultaneously or sequentially rather than operating as mutually exclusive processes. PANoptosis is an inflammation-associated form of RCD characterized by the coordinated activation of molecular components of pyroptosis, apoptosis, and necroptosis [55,68]. This process is mediated by multiprotein complexes termed PANoptosomes, which may comprise sensors, adaptors, caspases, and RIP kinases, with their composition varying according to the initiating stimulus [21].

PANoptosis is particularly relevant in inflammatory contexts, where activation of one cell death pathway may be modulated or amplified by another. Caspase-8 represents an important regulatory node between apoptosis and necroptosis, promoting apoptotic signaling while restricting RIPK1–RIPK3–MLKL-dependent necroptosis. Crosstalk between apoptosis and pyroptosis may also occur through gasdermin activation, while necroptotic signaling can promote inflammasome activation under specific conditions. At the same time, the simultaneous presence of apoptotic, pyroptotic, and necroptotic markers does not by itself establish PANoptosis. Evidence should demonstrate coordinated activation of these pathways within the same biological context and, ideally, functional interactions among their molecular components [21,69].

Crosstalk, however, extends beyond the three RCD pathways incorporated into PANoptosis. Mitochondrial damage can activate apoptotic signaling while disrupting redox homeostasis and increasing susceptibility to ferroptosis [70]. Similarly, OS can modulate inflammasome activity, autophagy can influence ferroptosis by regulating intracellular iron and damaged organelles, and metabolic alterations can modify susceptibility to both ferroptosis and cuproptosis. RCD pathways may also interact compensatorily, such that inhibition or failure of one death program can redirect cellular signaling toward an alternative execution mechanism [15,19,65]. This possibility has important therapeutic implications, because inhibition of a single RCD pathway may alter rather than completely prevent cell death; however, direct evidence for such compensatory pathway switching in reproductive tissues remains limited [2,24]. Thus, the predominant RCD phenotype is shaped by the initiating insult, cellular metabolic state, antioxidant capacity, inflammatory signaling, and availability of the relevant execution machinery [71,72]. This interconnected network is particularly relevant to reproductive biology, where redox, metabolic, mitochondrial, and immune processes undergo dynamic physiological changes.

The molecular indicators, confirmatory approaches, and functional validation strategies used to distinguish RCD-associated changes from experimentally confirmed cell death are summarized in Table 1.

## 4. RCD in Reproductive Physiology

Reproductive tissues are characterized by continuous cycles of proliferation, differentiation, remodeling, and cell elimination. Thus, RCD is not restricted to disease but also contributes to reproductive homeostasis. However, different RCD mechanisms have distinct levels of physiological relevance [2]. Apoptosis has a well-established role in physiological cell elimination, whereas autophagy predominantly contributes to cellular homeostasis, differentiation, and quality control and should not be equated with autophagy-dependent cell death. In contrast, the physiological roles of ferroptosis, pyroptosis, necroptosis, cuproptosis, and PANoptosis remain insufficiently established in reproductive biology and should presently be regarded as proposed or context-dependent rather than established components of normal reproductive homeostasis [37]. This distinction is important because the presence of RCD-associated molecules in reproductive tissues does not necessarily indicate activation of a specific cell death pathway. Both established physiological functions and potential roles of emerging RCD pathways are discussed in the following sections.

### 4.1. Gametogenesis and Folliculogenesis

In both male and female gametogenesis, cell survival and death must be tightly regulated. Since the production of germ cells in the testes substantially exceeds the number that ultimately complete differentiation, selective cell death is required to preserve the proper balance between developing germ cells and supporting Sertoli cells [73,74,75]. In this context, apoptosis is the most extensively studied RCD modality. During spermatogenesis, excess or damaged germ cells can be eliminated through the intrinsic apoptotic pathway, involving mitochondrial signaling, BCL-2 family proteins, and caspase activation. Germ cell apoptosis can also occur through extrinsic signaling via death receptors [9,75].

Autophagy also contributes to male germ cell development; however, this function must be distinguished from autophagy-dependent cell death. Autophagy-related mechanisms contribute to organelle and cytoplasmic remodeling during spermatogenesis and spermiogenesis, including mitochondrial quality control and the formation of specialized sperm structures [65,76]. In this case, autophagy is more closely associated with differentiation and homeostasis than with the execution of cell death.

Cell death contributes to follicular selection and atresia in the ovary. While only a small fraction of primordial follicles ultimately reach ovulation, the majority undergo atresia during follicular development. Cell death in granulosa cells represents a key process in follicular atresia and is modulated by the complex interplay between survival factors, gonadotropins, growth factors, and pro- and anti-apoptotic proteins of the BCL-2 family [77,78]. Oocyte survival is also influenced by mitochondrial function and apoptotic signaling, particularly during the establishment and progressive depletion of the ovarian reserve [79].

Recent evidence suggests that follicular and oocyte physiology may also be influenced by redox-sensitive RCD pathways. Oocytes and granulosa cells are metabolically active and depend on tightly regulated ROS production, antioxidant defenses, iron homeostasis, and mitochondrial function [80,81]. These characteristics may create susceptibility to ferroptotic injury when redox homeostasis is disrupted. However, the physiological role of ferroptosis in follicular selection remains poorly understood and is less well characterized than that of apoptosis. The physiological relevance of pyroptosis, necroptosis, and cuproptosis during gametogenesis also remains largely unresolved.

### 4.2. Fertilization and Early Embryonic Development

During fertilization and preimplantation development, cellular homeostasis must be maintained despite extensive metabolic and developmental remodeling. During the rapid progression from cleavage to blastocyst formation, gamete-derived molecular components contribute to embryonic development and survival [82,83].

As the embryo progresses toward the blastocyst stage, apoptosis occurs during normal preimplantation development and contributes to embryonic cell quality control [84]. Apoptosis may facilitate the selective elimination of damaged or developmentally compromised blastomeres, thereby supporting embryonic integrity, whereas excessive apoptosis is associated with reduced developmental competence. Therefore, the biological impact depends on the extent and timing of this process.

Autophagy plays an important role in supporting the oocyte-to-embryo transition and early embryonic development. The autophagic process contributes to the remodeling of intracellular contents and supplies substrates required for embryonic development [85,86,87,88]. Fertilization is accompanied by extensive degradation and turnover of maternally derived cellular components. Although mitophagy contributes to the preservation of mitochondrial quality, mitochondrial turnover is tightly regulated during the early stages of embryogenesis because mitochondrial function is essential for cellular energy metabolism [89,90].

The susceptibility of male and female gametes and embryos to OS and lipid peroxidation suggests a potential role for ferroptosis in these cells. However, the mere presence of OS does not establish the involvement of ferroptosis. Enhanced ROS production or lipid peroxidation alone is insufficient to establish ferroptosis; evidence of iron-dependent lipid peroxidation and involvement of ferroptosis-specific regulatory machinery is also required [1,38,91].

### 4.3. Endometrial Remodeling and Embryo Implantation

The endometrium constitutes one of the most dynamically remodeled adult tissues. Throughout the menstrual cycle, coordinated changes in proliferation, differentiation, immune cell recruitment, vascular remodeling, decidualization, and tissue breakdown dynamically regulate cell survival and death. RCD contributes to this remodeling, with its biological effects depending on the stage of the menstrual cycle and the specific cellular compartment [92,93,94].

Reduced ovarian steroid support during the late secretory and menstrual phases in the absence of pregnancy promotes tissue remodeling, inflammation, and increased cell death. Apoptosis plays a role in the removal of epithelial and stromal cells of the endometrium, and processes such as proteolysis and inflammation contribute to tissue breakdown and regeneration [95]. Autophagic activity also varies across the menstrual cycle and has been implicated in cellular adaptation during decidualization and endometrial remodeling, although its specific roles depend on cell type and the hormonal environment [96].

Implantation requires a distinct balance between embryo adhesion and trophoblast invasion, preservation of tissue integrity, and tightly regulated maternal immune responses [97]. Decidual stromal cells undergo extensive metabolic and phenotypic reprogramming, and excessive activation of cell death pathways may compromise the formation and maintenance of a functional decidual layer. Conversely, the regulated clearance of senescent cells may contribute to tissue remodeling and quality control [98].

Inflammation-associated RCD processes are particularly relevant at the maternal–embryo interface, where a controlled inflammatory environment contributes to implantation. Although inflammasome assembly and inflammatory cytokine signaling contribute to endometrial physiology, inflammasome activation should be distinguished from pyroptotic cell death [2,99,100]. Likewise, the presence of proteins associated with ferroptosis, necroptosis, or PANoptosis does not establish the physiological relevance of these pathways in the endometrium; their involvement in reproductive disorders is discussed separately below [101,102].

### 4.4. Placentation and the Maternal–Fetal Interface

Placental development is associated with controlled proliferation, differentiation, fusion, invasion, and turnover of trophoblasts. These processes occur within a dynamic microenvironment characterized by changes in oxygen availability and complex interactions among maternal immune cells, the vasculature, and fetal trophoblasts [103,104]. Consequently, RCD contributes to placental development, and its dysregulation may impair placental structure and function.

Apoptosis regulates trophoblast cell number by eliminating damaged or senescent cells and remains the most extensively studied RCD mechanism in placental biology. During placental development, apoptosis contributes to trophoblast turnover and the elimination of damaged cells, including within the syncytiotrophoblast [2,105,106]. Physiological trophoblast turnover releases cellular and extracellular material into the maternal circulation, whereas the abundance and molecular composition of these products may be altered in pregnancy complications [107,108].

Autophagy constitutes an important adaptive mechanism in trophoblasts [109]. Placental cells are exposed to fluctuations in oxygen and nutrient availability and undergo substantial metabolic adaptation; under these conditions, autophagic recycling may support cellular survival by maintaining energy balance and organelle quality [110]. As in other cell types, increased autophagic activity in placental cells does not necessarily indicate autophagy-dependent cell death.

Pregnancy-related changes in oxygen availability, mitochondrial function, iron homeostasis, lipid metabolism, and immune signaling may influence redox- and inflammation-associated RCD pathways in the placenta. These changes may create conditions that favor the activation of necroptotic, pyroptotic, and ferroptotic pathways [111,112]. However, the physiological roles of these pathways in normal human placentation remain less well characterized than their involvement in placental pathology. This distinction is particularly important in preeclampsia, fetal growth restriction, and other placental disorders, in which oxidative and inflammatory stress may shift adaptive signaling toward cellular injury and death [113].

### 4.5. Reproductive Aging

Reproductive aging is characterized by a progressive decline in the number and quality of reproductive cells and tissues, accompanied by changes in the regulation of RCD pathways. This process is associated with reduced oocyte competence and progressive depletion of the ovarian reserve, primarily through follicular atresia [114,115]. Although reproductive aging in males is less abrupt, it is nonetheless marked by alterations in testicular activity, sperm quality, genome stability, mitochondrial function, and oxidative balance [116].

Mechanisms beyond apoptosis-mediated follicular loss also contribute to reproductive aging. Age-related mitochondrial dysfunction can lead to reduced ATP production, impaired cellular stress resilience, and increased ROS generation [117,118,119]. Reduced antioxidant capacity and changes in iron and lipid metabolism may increase susceptibility to ferroptotic injury. Damaged macromolecules may accumulate as a result of reduced autophagy and mitophagy efficiency, which can accompany aging [120,121].

This highlights an important distinction between the physiological functions of RCD and their dysregulation during aging [122]. Cell elimination through physiological processes is an important part of reproductive homeostasis, whereas aging-related cellular alterations may modify the thresholds for activation of different RCD pathways [123]. Ferroptosis and other RCD pathways may therefore contribute to reproductive aging, although their specific roles in humans remain unclear [120].

Importantly, the biological significance of RCD is highly cell-type dependent. In oocytes, inappropriate cell death may contribute to depletion of the finite germ-cell pool, whereas in granulosa cells, apoptosis is an integral component of follicular selection and atresia but may become detrimental when excessive or dysregulated [11,12,78]. In mature spermatozoa, their limited transcriptional and translational capacity requires particular caution when interpreting canonical RCD markers, while oxidative membrane and DNA damage can substantially impair sperm function [10]. In decidual cells, RCD occurs within the dynamic processes of decidualization, immune adaptation, and endometrial remodeling [93], whereas in trophoblasts, regulated cell survival and death contribute to placental development and turnover, with excessive or inflammatory RCD potentially impairing trophoblast function and placentation [97,104,105,106]. Thus, the biological consequences of RCD depend on the reproductive cell type, developmental stage, and extent of pathway activation.

Overall, substantial evidence supports the physiological role of apoptosis and the predominantly homeostatic function of autophagy across different stages of reproduction. However, rather than being established components of normal reproductive physiology, ferroptosis, pyroptosis, necroptosis, cuproptosis, and PANoptosis are currently better characterized as cellular responses associated with stress and disease. This distinction should be carefully considered when interpreting the growing body of evidence on RCD in reproductive disorders.

## 5. RCD in Reproductive Disorders

The contribution of RCD to reproductive disease depends not only on the activation of a particular death program but also on its magnitude, timing, cellular localization, and interaction with the surrounding microenvironment. Dysregulated RCD may contribute to germ-cell loss, impaired follicular development, persistence of ectopic endometrial cells, implantation defects, and placental injury [124,125,126,127].

At the same time, the level of scientific evidence varies greatly across RCD pathways and reproductive disorders. Apoptosis is supported by extensive experimental and human evidence, whereas evidence for ferroptosis and pyroptosis has expanded in recent years [124,126,127,128]. In contrast, the involvement of necroptosis, cuproptosis, and PANoptosis in reproductive disorders remains supported by relatively limited experimental and human data, although direct evidence of necroptosis has been reported in specific reproductive contexts, including preeclampsia and male infertility [128,129]. Therefore, this section places particular emphasis on the strength of experimental and clinical evidence supporting the involvement of individual RCD pathways.

**Figure 2:**
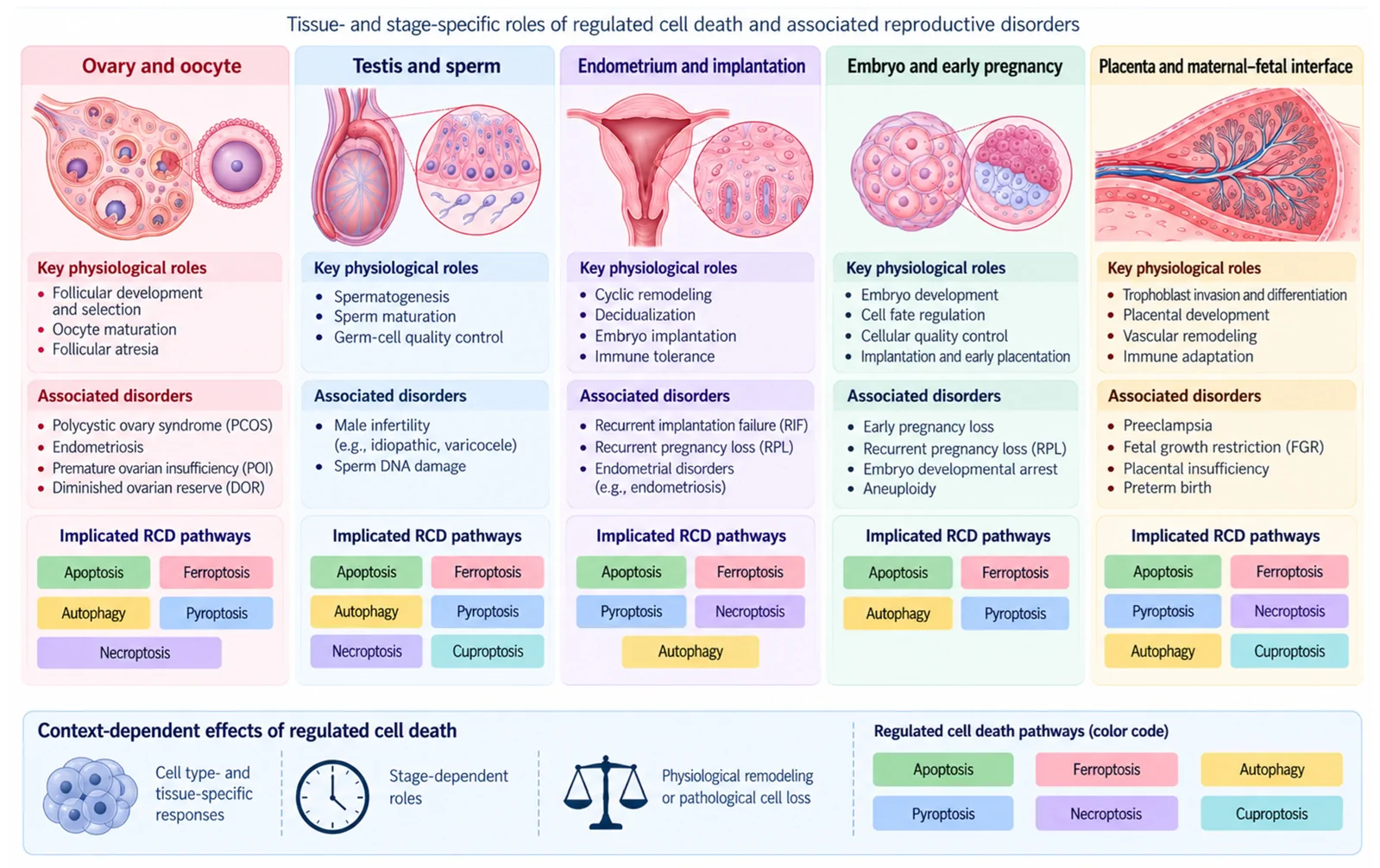
Regulated cell death in reproductive physiology and disease. Overview of the tissue- and stage-specific roles of RCD across major reproductive compartments, associated reproductive disorders, and implicated RCD pathways. The biological effects of individual RCD pathways are context-dependent and vary according to cell type, reproductive stage, and pathological setting. Colors distinguish the major RCD pathways represented in the figure. This figure is an original illustration created by the authors using ChatGPT (OpenAI) and was not reproduced or adapted from previously published material.

The involvement of individual RCD pathways varies substantially across reproductive tissues, cell types, and disease contexts, reflecting their distinct physiological functions and their context-dependent contribution to reproductive pathology (Figure 2).

### 5.1. Female Infertility and Ovarian Disorders

#### 5.1.1. PCOS

PCOS involves metabolic and reproductive disturbances such as anovulation, hyperandrogenism, abnormal follicle growth, insulin resistance, and in many women, low-grade inflammation and OS, which may influence RCD pathways in granulosa cells and the ovarian milieu [130,131].

Altered granulosa cell apoptosis has been considered a potential contributor to disrupted follicular growth in PCOS. Changes in pro- and anti-apoptotic signaling and caspase activity have been described in human granulosa cells and animal models of PCOS [81,132]. However, PCOS cannot be characterized by uniformly increased or decreased ovarian apoptosis, as the direction and biological significance of apoptotic changes vary with follicular stage and the experimental model used [81].

More recent studies have increasingly focused on ferroptosis. Experimental models of PCOS have demonstrated alterations in iron metabolism, lipid peroxidation, glutathione homeostasis, and the expression of ferroptosis-regulatory proteins [133,134]. Granulosa cells are of particular interest because of their critical role in oocyte maturation and their susceptibility to redox and metabolic disturbances. Granulosa cell death and ovarian phenotype in PCOS models have been shown to be modulated by GPX4-, SLC7A11-, and iron-dependent redox pathways [133,134,135].

Functional manipulation of these pathways provides stronger mechanistic evidence than changes in protein expression alone; nevertheless, definitive evidence establishing ferroptosis as a major cell-death mechanism in human PCOS remains lacking [81].

PCOS has also been linked to inflammation mediated by pyroptosis and inflammasome signaling. Increased NLRP3-associated inflammatory signaling has been reported in the ovaries and metabolically active tissues, while some studies suggest that gasdermin-mediated inflammatory cell death may contribute to granulosa cell dysfunction [24,136,137]. However, increased levels of NLRP3, caspase-1, IL-1β, and IL-18 in the absence of evidence of gasdermin-dependent execution indicate inflammasome activation rather than definitive pyroptotic cell death. Similarly, bioinformatic studies have identified PANoptosis-related molecular signatures in PCOS, although recent experimental studies have begun to provide functional evidence implicating PANoptotic signaling in granulosa-cell dysfunction and ovulatory impairment. Nevertheless, further validation is required to establish the extent and clinical relevance of PANoptosis in human PCOS [138,139,140,141].

Overall, evidence for non-apoptotic RCD in PCOS is strongest for ferroptosis and inflammation-related pyroptotic pathways, although their significance in human disease remains uncertain. Notably, RCD alterations in PCOS should not uniformly be interpreted as primary drivers of disease. Functional studies support a potential direct contribution of ferroptosis-related granulosa-cell injury to ovarian dysfunction [134,135], whereas some apoptotic, pyroptotic, and inflammasome-associated changes may occur secondarily to the metabolic disturbances, OS, and chronic inflammation characteristic of PCOS [130,131,132,136,137,138]. Thus, particularly in human studies, the direction of causality between RCD dysregulation and the broader PCOS phenotype remains incompletely established.

#### 5.1.2. Endometriosis

Endometriosis represents a distinct context in which resistance to cell death, rather than excessive cell loss, may contribute to disease persistence. To establish ectopic lesions, displaced endometrial cells must survive detachment and retrograde transport, adhere and implant at ectopic sites, and adapt to the peritoneal microenvironment.

Alterations in BCL-2 family proteins, death receptor signaling, and caspase activation have been observed in the eutopic and ectopic endometrium of women with endometriosis. Impaired apoptotic clearance may promote the persistence of endometrial cells, although the extent of this alteration may vary according to lesion type and menstrual cycle phase [125]. Conversely, established lesions reside in a pro-inflammatory and oxidative environment that could induce other RCD mechanisms.

Ferroptosis is particularly relevant to endometriosis because recurrent bleeding within ectopic lesions promotes iron accumulation, heme toxicity, OS, and lipid peroxidation, creating a microenvironment conducive to ferroptotic stress. Human endometriotic tissues exhibit alterations in ferroptosis-related regulators, including GPX4, ACSL4, and transferrin receptor 1 [142]. Experimental studies have provided stronger functional evidence by demonstrating that ferroptosis induction can alter endometriotic stromal cell survival and promote lesion regression in experimental models [143]. Induction of ferroptosis may therefore offer a therapeutic strategy for targeting apoptosis-resistant ectopic lesions; however, non-selective ferroptosis induction could also affect healthy reproductive tissues. Ovarian and eutopic endometrial cells may be susceptible to disturbances in iron and lipid-redox homeostasis, raising the possibility of impaired follicular integrity, oocyte competence, or endometrial function [127,142]. Therefore, cell-specific targeting and careful evaluation of reproductive toxicity will be essential before ferroptosis-inducing strategies can be translated to the treatment of endometriosis [142,143].

Pyroptosis and inflammasome signaling represent another potential link between RCD and the chronic inflammatory environment of endometriosis. NLRP3 inflammasome activation has been reported in experimental models and human endometriosis; however, evidence of complete gasdermin-mediated pyroptotic execution remains limited. Necroptotic signaling has also been investigated; however, evidence in this area remains considerably more limited than that for apoptotic and ferroptotic pathways [3,144,145,146].

The coexistence of oxidative damage, inflammatory signaling, and apoptosis resistance emphasizes why RCD in endometriosis cannot be explained by a single directional change in cell death activity. Distinct RCD mechanisms may operate across different cell types, with the same pathway contributing to tissue injury while also representing a potential therapeutic target.

#### 5.1.3. Premature Ovarian Insufficiency and Diminished Ovarian Reserve

POI is characterized by loss of ovarian function before the age of 40 years and may result from genetic, autoimmune, iatrogenic, metabolic, or environmental factors. Accelerated follicular depletion and impaired follicular function make RCD particularly relevant to POI pathophysiology [147].

Excessive oocyte and granulosa cell death is among the most extensively studied mechanisms of follicular depletion in experimental models of ovarian injury [78]. Chemotherapy and other gonadotoxic exposures can induce stress-mediated apoptosis through DNA damage and altered intrinsic apoptotic signaling. However, POI cannot be attributed to a single apoptotic mechanism, as the initiating insult and affected cell populations vary across different etiologies [148,149].

Ferroptosis has also been proposed as a mediator of ovarian injury because of its dependence on iron metabolism, OS, and lipid peroxidation. Altered lipid peroxidation and GPX4/SLC7A11 signaling have been reported in models of chemically induced ovarian insufficiency, in which modulation of ferroptosis-related pathways can influence ovarian injury and function [133,150,151,152,153]. Although such experimental studies provide mechanistic insight into ferroptosis in induced animal models, extrapolation to spontaneous human POI requires caution. Human studies remain limited, and most rely on indirect assessments. Experimentally induced ovarian injury models reproduce defined gonadotoxic or chemical insults and therefore do not capture the heterogeneous etiologies of human POI [147,148,149]. Accordingly, mechanistic evidence obtained in these models should not be interpreted as demonstrating the same RCD pathway in patients without direct human validation [150,151,152,153].

Pyroptotic signaling may also contribute to granulosa cell injury in POI, with experimental evidence implicating NLRP3-mediated pyroptosis and showing that its modulation can influence ovarian reserve function [154]. However, evidence remains substantially more limited than that available for apoptosis and ferroptosis.

Autophagy and mitophagy have context-dependent roles in maintenance of the ovarian reserve. Altered autophagic flux may impair mitochondrial and protein homeostasis, whereas basal autophagy supports oocyte and granulosa cell homeostasis [155,156]. Conversely, cell death may coexist with autophagy activation during severe stress. Therefore, increased autophagic markers alone do not indicate autophagy-dependent cell death unless a causal role of the autophagic machinery in cell death is demonstrated.

DOR, although clinically characterized by reduced ovarian reserve and altered ovarian response, should not be considered mechanistically synonymous with POI. Specific RCD mechanisms in DOR remain understudied. Recent human evidence has identified ferroptosis-related and sterol-biosynthesis alterations in granulosa cells from patients with DOR [157]; however, such molecular associations should not be considered equivalent to functional demonstration of ferroptotic cell death.

### 5.2. Male Infertility

Male germ cells and mature spermatozoa are particularly susceptible to disturbances in redox homeostasis. Spermatogenesis requires high rates of cellular proliferation and differentiation, while sperm plasma membranes contain abundant polyunsaturated fatty acids and mature sperm possess limited cytoplasmic antioxidant and repair capacity. RCD dysregulation can therefore affect germ-cell survival during spermatogenesis and the functional integrity of sperm after their release [158,159].

Excessive germ-cell apoptosis has been described across several causes of male infertility, including testicular heat stress, hormonal disturbance, toxicant exposure, and varicocele. Increased apoptotic markers in ejaculated sperm have also been associated with impaired conventional semen parameters and DNA integrity [160,161,162]. However, mature sperm display distinctive cellular architecture and may exhibit apoptosis-like molecular features without undergoing the complete canonical apoptotic program observed in somatic cells. Individual apoptotic markers in ejaculated sperm should therefore be interpreted with caution [163].

Ferroptosis is particularly relevant because iron-dependent lipid peroxidation converges with established mechanisms of oxidative sperm damage. Experimental disruption of GPX4 profoundly impairs male fertility, highlighting its role in controlling phospholipid peroxidation and supporting specialized functions during sperm development [164,165]. More recent human studies have identified alterations in ferroptosis-associated markers in semen from men with different reproductive pathologies and evidence of ferroptosis-related changes in spermatozoa from infertile men with bilateral varicocele [128,166]. Nevertheless, conventional lipid peroxidation in sperm should not automatically be classified as ferroptosis. Evidence of iron dependence and engagement of ferroptosis-regulatory machinery is required to distinguish ferroptotic cell death from nonspecific oxidative membrane damage.

More broadly, the detection of ROS, lipid peroxidation, DNA damage, or individual RCD-associated markers in mature spermatozoa does not establish execution of a specific RCD pathway. Stronger mechanistic evidence requires pathway-specific molecular or biochemical features together with functional demonstration of pathway dependency through inhibition, rescue, or, where experimentally feasible, genetic manipulation.

Pyroptotic and necroptotic mechanisms have been investigated primarily in inflammatory or experimentally induced testicular injury. Evidence from infertile men with bilateral varicocele has also demonstrated concurrent alterations in markers associated with ferroptosis, pyroptosis, and necroptosis in spermatozoa [128]. Activation of inflammasome–gasdermin signaling may amplify local inflammation and compromise Sertoli or germ-cell function, while RIPK3–MLKL signaling has been implicated in selected models of testicular damage. The extent to which these pathways independently contribute to common forms of human male infertility remains less well established.

Thus, apoptosis remains the most firmly established RCD mechanism in male infertility, while evidence supporting ferroptosis is increasing. Evidence for other RCD modalities remains limited to specific etiologies and experimental models.

### 5.3. Implantation Failure and Recurrent Pregnancy Loss

Successful implantation and pregnancy require tightly coordinated regulation of cell survival, proliferation, differentiation, and death across embryonic and maternal compartments, together with appropriate immune adaptation [167,168]. Dysregulation of these processes may impair endometrial receptivity, decidualization, trophoblast invasion, or maternal immune tolerance. However, given the heterogeneity of RIF and RPL, RCD alterations identified in specific patient populations should not be generalized to all affected individuals [169].

The definition of RIF is not uniform across the cited literature, with studies differing in the number of unsuccessful embryo transfers or implantation attempts used for inclusion and in the characteristics of transferred embryos. Consequently, RCD-associated findings should be interpreted according to the specific RIF criteria applied in each study. Embryo-related factors, including developmental competence and chromosomal status, may independently influence implantation, while maternal age, endometrial and uterine characteristics, metabolic and immune factors, and treatment-related variables may further confound associations between RCD-related alterations and implantation failure [168,169]. These sources of heterogeneity limit causal interpretation and comparison across RIF studies.

In RIF, changes in apoptotic pathway signaling have been observed in the endometrium and have been associated with decidualization defects, inflammation, and abnormal turnover [170]. Alterations in endometrial hypoxia-related signaling, angiogenesis, and apoptosis have also been reported in women with RIF. Similarly, ferroptosis pathways have gained interest owing to the requirement for regulated redox and lipid metabolism in endometrial receptivity. Transcriptomic analyses have identified ferroptosis-related signatures and candidate biomarkers in patients with RIF [171]. However, neither gene set enrichment nor ferroptosis-related transcriptional signatures alone can establish ferroptotic cell death in the receptive endometrium.

RPL provides an additional context in which RCD at the maternal–fetal interface may be relevant. Increased trophoblast apoptosis has been reported in pregnancy loss and may compromise trophoblast viability and development, with adverse consequences for placental function [2,172,173]. Inflammation, OS, and mitochondrial dysfunction may exacerbate trophoblast injury. Recent experimental studies have also implicated ferroptosis-related mechanisms in pregnancy loss, including altered trophoblast ferroptosis regulation and disruption of mechanisms that normally confer ferroptosis resistance at the maternal–fetal interface [174,175,176]. Alterations in NLRP3, caspase-1, gasdermins, and inflammatory cytokines have been reported in RPL, suggesting potential involvement of pyroptotic signaling. Experimental evidence further indicates that environmental toxicant exposure can induce trophoblast pyroptosis through the lnc-HZ14/ZBP1/NLRP3 axis and promote pregnancy loss [126].

A common limitation in both RIF and RPL is that tissue samples are frequently obtained after implantation or pregnancy failure has already occurred. Consequently, observed RCD alterations may represent either causal mechanisms or secondary responses to tissue injury and pregnancy failure. Prospective and mechanistically oriented studies are therefore required to establish temporal and causal relationships.

### 5.4. Placental and Pregnancy Disorders

Placental disorders provide a relevant context for investigating stress-associated RCD because they involve OS, mitochondrial dysfunction, inflammation, altered blood flow, and metabolic disturbances. Among these conditions, preeclampsia and FGR have received substantial attention, whereas evidence for specific RCD pathways in GDM and preterm birth remains comparatively limited [177].

Increased trophoblastic cell death and impaired syncytiotrophoblast reorganization are features of preeclampsia. Ferroptosis has emerged as a potential mechanism in preeclampsia, particularly in the context of placental OS and impaired antioxidant capacity [178,179]. Alterations in lipid peroxidation, iron metabolism, and ferroptosis-regulatory molecules, including GPX4, have been reported in placentas from patients with preeclampsia, while functional modulation of ferroptosis-related pathways can influence trophoblast viability and preeclampsia-associated phenotypes in experimental models [112,180]. Although the contribution of ferroptosis relative to other forms of trophoblast cell death remains unclear, current evidence suggests a potential link between placental redox imbalance and ferroptotic signaling in preeclampsia [181,182].

Pyroptosis may provide an additional link between inflammation and placental cell injury. Inflammasome activation and gasdermin-dependent signaling have been reported in placental tissues from patients with preeclampsia and in experimental animal models [181,183]. Alterations in RIPK1/RIPK3/MLKL signaling have also been reported, suggesting potential involvement of necroptosis. These mechanisms may occur concurrently rather than independently, particularly in the placental environment characterized by hypoxia, OS, and inflammation [184,185].

FGR is a heterogeneous condition with maternal, fetal, and placental etiologies, several of which involve overlapping mechanisms of OS, inflammation, and placental dysfunction. Placental insufficiency-associated FGR has been linked to OS, mitochondrial dysfunction, and increased placental apoptosis, whereas the involvement of ferroptosis, pyroptosis, and necroptosis remains less well established [186,187]. Therefore, the specific placental pathology should be considered when interpreting the involvement of these RCD pathways.

In GDM, hyperglycemia, insulin resistance, OS, and placental inflammation may alter trophoblast survival and autophagic activity [188,189]. Experimental studies suggest the involvement of ferroptosis and pyroptosis, although the supporting evidence remains more limited than in preeclampsia. Inflammation-induced RCD has also been studied in infectious and inflammatory preterm birth, in which inflammasomes and gasdermin signaling have been implicated as potential mediators of tissue injury [178,190].

Collectively, placental disorders illustrate how multiple RCD pathways may converge within a shared environment of oxidative, metabolic, and inflammatory stress. Evidence is strongest for dysregulated apoptosis, while substantial mechanistic evidence also supports the involvement of ferroptosis and pyroptosis, particularly in preeclampsia. An important unresolved question is whether these pathways are activated independently or through coordinated RCD mechanisms such as PANoptosis.

Representative primary studies investigating distinct RCD pathways across reproductive and pregnancy-related disorders are summarized in Table 2, with emphasis on the experimental model, molecular findings, reproductive outcomes, and strength of evidence.

**Table 2:** Representative primary studies investigating RCD in reproductive disorders.

Author, Year	Condition	Study Design/Population or Model	RCD Pathway	Main Molecular Findings	Main Reproductive Findings	Evidence Type/Strength
Zhu et al., 2026 [191]	PCOS	DHEA-induced PCOS mouse model; GC-specific *Gpx4* knockout; pharmacological and multi-omics analyses	Ferroptosis	MBD2-mediated epigenetic repression of *Gpx4* promoted GC ferroptosis; MBD2/DNMT inhibition restored *GPX4* and reduced ferroptosis, whereas RSL3 or *Gpx4* deletion abolished protection	*Gpx4* loss impaired follicular maturation and corpora lutea formation and aggravated PCOS; MBD2 inhibition alleviated ovarian dysfunction	Animal model + primary mouse granulosa cells|Functionally validated: genetic and pharmacological manipulation with functional rescue
Huang et al., 2025 [135]	PCOS	PCOS patient samples; transcriptomic/metabolomic analyses; KGN GC experiments; SCM-198 intervention	Ferroptosis	PCOS was associated with GC ferroptosis and reduced follicular-fluid butyrate; butyrate suppressed GC ferroptosis through SLC7A11/TXNRD1/GPX4	SCM-198 improved ovarian function, hormonal abnormalities and insulin resistance while reducing GC ferroptosis	Human samples + granulosa cell model + animal model|Functionally validated: pharmacological intervention targeting the SLC7A11/GPX4 axis with functional rescue of ferroptosis and ovarian dysfunction
Meresman et al., 2000 [125]	Endometriosis	Human case–control study; eutopic endometrium from 14 women with endometriosis and 16 controls; TUNEL and IHC	Apoptosis	Spontaneous apoptosis was significantly reduced; Bcl-2 was increased in proliferative endometrium, while Bax showed cycle-dependent expression	Reduced apoptosis supports abnormal survival of endometrial cells and may contribute to persistence at ectopic sites	Patient endometrial tissue|Mechanistically supportive: direct assessment of apoptosis by TUNEL with Bcl-2/Bax expression, but observational and without functional validation
Li et al., 2021 [192]	Endometriosis	Primary stromal cells from 21 women with endometriosis and 11 controls; pharmacological/genetic experiments; mouse model	Ferroptosis	Erastin increased iron, ROS/lipid ROS and ferroptotic mitochondrial changes; ferroptosis inhibitors attenuated death and FPN manipulation bidirectionally regulated ferroptosis	EESCs were more susceptible to ferroptosis; erastin induced regression of ectopic lesions in mice	Primary human cells + animal model|Functionally validated: ferroptosis-specific pharmacological rescue and FPN genetic manipulation, with in vivo lesion regression
Mielke Cabello et al., 2024 [142]	Endometriosis	Retrospective case–control study; 38 women with endometriosis and 18 controls; GPX4, ACSL4 and TfR1 IHC	Ferroptosis-associated alterations	Stromal GPX4 was significantly reduced; marker expression differed between stromal and epithelial compartments	Findings support altered ferroptosis-associated profiles but do not establish ferroptotic execution or disease causality	Patient-derived endometriosis tissue|Associative: ferroptosis-regulator expression without functional demonstration of ferroptotic cell-death execution
Yu et al., 2025 [157]	DOR	Human comparative study; GCs and follicular fluid from women with DOR and normal ovarian reserve; RNA-seq, metabolomics and cellular analyses	Ferroptosis-associated alterations/apoptosis	Increased apoptosis and iron staining, reduced GPX4/SLC7A11 and ferroptosis-like mitochondrial morphology; altered sterol metabolism and FSHR signaling	GC proliferation was reduced and follicular-fluid androgen levels increased	Patient-derived granulosa cells + follicular fluid|Mechanistically supportive: convergent molecular, metabolic, and morphological ferroptosis-associated features, but without pathway-specific functional rescue or genetic validation
Moretti et al., 2024 [166]	Male infertility (varicocele/urogenital infection)	Human observational study; 28 infertile men and 19 fertile controls; seminal iron/redox markers and sperm ACSL4/GPX4	Ferroptosis-associated alterations	Ferritin/transferrin correlated with F2-isoprostanes and ACSL4; ACSL4 was elevated in infertile groups, whereas GPX4 was unchanged	Ferritin and ACSL4 correlated negatively with semen parameters	Human semen/sperm samples|Associative: ferroptosis-related biochemical and molecular alterations, but without functional demonstration of ferroptotic cell-death execution
Sun et al., 2023 [128]	Male infertility (bilateral varicocele)	Human comparative study; 26 infertile men and 20 healthy controls; semen functional, redox and RCD-marker analyses	Ferroptosis-, pyroptosis- and necroptosis-associated alterations/apoptosis	ROS/iron increased and antioxidant defenses decreased; ferroptosis-related transcripts decreased, while RIPK1/RIPK3 and GSDME/HSP90 increased	Varicocele was associated with impaired sperm functional parameters and altered apoptosis	Human sperm samples|Associative: multiple ferroptosis-, pyroptosis-, and necroptosis-associated molecular and biochemical alterations, but without pathway-specific functional rescue or genetic validation
Yu et al., 2019 [193]	RIF	Controlled human study; peri-implantation endometrium from 20 women with RIF and 10 fertile controls; endometrial-injury component	Apoptosis	Endometrial apoptosis, HIF-1α and microvessel density were reduced; injury increased HIF-1α and microvessel density but did not significantly alter apoptosis	Reduced peri-implantation apoptosis was associated with RIF alongside impaired HIF-1α expression and angiogenesis	Patient endometrial tissue|Mechanistically supportive: direct assessment of apoptosis and angiogenesis, but without functional validation of apoptosis as a causal mechanism in RIF
Zhou et al., 2025 [171]	RIF	GEO GSE106602 analysis using WGCNA/FerrDB; validation in endometrial samples and implantation-outcome analysis	Ferroptosis-related gene signature	Fifty-four ferroptosis-related genes were identified; 23 hub genes were differentially expressed, and six genes correlated with implantation outcomes	Six-gene ImplantScore was lower in failed implantation and showed an AUC of 0.891 for outcome discrimination	Bioinformatic/transcriptomic analysis + patient endometrial tissue validation|Associative: ferroptosis-related gene signature with implantation-outcome association, but without functional demonstration of ferroptotic cell-death execution
Wang et al., 2023 [126]	Recurrent miscarriage/BaP-associated miscarriage	Human RM/control villous tissues; BPDE-exposed human trophoblasts; BaP-exposed mouse miscarriage model; genetic manipulation	Pyroptosis	BPDE activated NLRP3/caspase-1/GSDMD; *lnc-HZ14* promoted ZBP1/NLRP3-dependent pyroptosis through IRF1, METTL3-mediated m6A regulation and ZBP1/NLRP3 interaction; pathway knockdown attenuated pyroptosis	Pyroptosis-related changes were increased in RM villi; BaP induced placental pyroptosis and miscarriage, whereas *Nlrp3* knockdown reduced pyroptosis and miscarriage	Patient placental tissue + human trophoblast cell model + animal model|Functionally validated: pyroptotic execution with genetic manipulation and in vivo functional rescue
Qin et al., 2024 [176]	Recurrent miscarriage	Human RM/control villous tissues and GSE76862 data; RSL3-treated HTR8 trophoblasts; ALKBH5 overexpression and transcriptomic integration	Ferroptosis-associated trophoblast injury	RM villi showed reduced global m6A and increased ALKBH5/FTL; ALKBH5 overexpression reduced RSL3-induced death and oxidative products, with FTL identified as a downstream ferroptosis-related target	Increased ALKBH5/FTL in RM cytotrophoblasts was associated with a potentially protective response against ferroptosis-related trophoblast injury	Patient placental tissue + trophoblast cell model|Mechanistically supportive: ALKBH5 manipulation and ferroptosis-associated changes, but without ferroptosis-specific functional rescue demonstrating pathway dependency
Zhu et al., 2025 [175]	Recurrent pregnancy loss/early pregnancy loss	Human RPL placental specimens; primary human CTBs/STBs and BeWo syncytialization model; LPS-induced mouse early-pregnancy-loss model	Ferroptosis	CTB-derived lactate was transported to STB through MCT1 and activated PI3K/AKT/mTOR signaling, increasing SCD1 and GPX4 and reducing lipid peroxidation and ferroptosis susceptibility; RPL placentas showed impaired lactate synthesis and increased ferroptosis biomarkers	Lactate administration reduced placental ferroptosis and rescued fetal loss in LPS-exposed mice, supporting a protective role of the trophoblast lactate shuttle in placental homeostasis	Patient placental tissue + primary human trophoblasts + trophoblast cell model + animal model|Functionally validated: defined metabolic mechanism with modulation of ferroptosis susceptibility and in vivo rescue of pregnancy loss

## 6. RCD in Gynecological Malignancies

Abnormal regulation of cell death is one of the hallmarks of cancer biology. Cancer cells are often resistant to apoptosis and, at the same time, undergo metabolic, oxidative, and inflammatory changes that may alter their sensitivity to other forms of RCD. In gynecological malignancies, increasing attention has focused on alternative RCD mechanisms, including ferroptosis, pyroptosis, necroptosis, autophagy-dependent cell death, and cuproptosis. However, the strength of evidence varies considerably across RCD modalities [25,194,195].

### 6.1. Ovarian Cancer

Ovarian carcinoma, especially high-grade serous ovarian carcinoma, is marked by high levels of genomic instability, metabolic flexibility, OS, and chemoresistance. Resistance to apoptosis contributes to cancer cell survival following chemotherapy and has prompted investigation of alternative RCD pathways [196,197,198].

Ferroptosis is among the most extensively investigated emerging RCD mechanisms in ovarian cancer. Ovarian cancer cells may exhibit increased dependence on iron metabolism, lipid metabolism, glutathione homeostasis, and antioxidant defenses, potentially increasing their susceptibility to ferroptosis [199,200,201]. Experimental studies have shown that inhibition of GPX4 or system Xc^−^ induces lipid peroxidation and ferroptotic cell death in ovarian cancer cells, whereas alterations in ACSL4, iron transport, and glutathione metabolism can modulate susceptibility to ferroptosis. This dependence on antioxidant defenses may increase the susceptibility of ovarian cancer cells to ferroptosis, supporting the investigation of ferroptosis induction as a potential therapeutic strategy [202,203].

The connection between ferroptosis and the tumor microenvironment also deserves attention. The tumor stroma, immune cells, hypoxia, nutrient availability, and lipid composition may influence susceptibility to ferroptosis and determine its effects on tumor progression. Lipid peroxidation products generated during ferroptosis may influence antitumor immune responses, suggesting that its effects are not restricted to the dying cell [204,205].

Likewise, pyroptosis has been identified as a possible approach for therapy in ovarian cancer. Gasdermin-mediated membrane permeabilization can induce rapid tumor cell lysis and the release of inflammatory mediators, potentially enhancing tumor immunogenicity [206]. However, the inflammatory consequences of pyroptosis are context-dependent and may promote either antitumor immunity or tumor-promoting inflammation.

Other RCD mechanisms, including necroptosis and autophagy-mediated pathways, have been explored, although their functional consequences are highly context-dependent. Necroptosis can serve as an alternative mechanism for targeting apoptosis-refractory tumor cells; nevertheless, sustained necroptotic activity and the release of DAMPs can also modify the tumor microenvironment [25,207]. Autophagy may support ovarian cancer cell survival under metabolic or therapeutic stress, whereas under specific experimental conditions, excessive or dysregulated autophagic activity may contribute to cell death. As in normal tissues, elevated autophagic flux does not necessarily imply autophagy-dependent cell death [208,209].

Cuproptosis has gained interest because of the metabolic heterogeneity of ovarian cancer. As cuproptosis involves mitochondrial respiration and lipoylation of tricarboxylic acid cycle proteins, susceptibility to this pathway may depend on the metabolic state of individual tumors [210,211]. However, current research on cuproptosis in ovarian cancer largely relies on gene-expression signatures, prognostic models, and computational patient stratification rather than direct functional demonstration of cuproptotic cell death.

### 6.2. Endometrial Cancer

Endometrial cancer is closely associated with hormonal and metabolic factors and frequently develops in the context of obesity, insulin resistance, inflammation, and dysregulated lipid metabolism. These features provide a strong biological rationale for the involvement of RCD pathways regulated by cellular metabolic state [212].

Apoptosis resistance contributes to endometrial cancer progression and therapeutic resistance, whereas ferroptosis has emerged as a potential alternative therapeutic target. Experimental studies have shown that modulation of GPX4, SLC7A11, iron homeostasis, and lipid peroxidation can influence the survival of endometrial cancer cells. Dysregulation of the PI3K/AKT/mTOR pathway, frequently observed in endometrial cancer, may also influence susceptibility to ferroptosis [213,214,215].

In endometrial cancer, pyroptosis-related signaling has also been investigated, particularly inflammasome activation and gasdermin expression. However, alterations in pyroptosis-related markers or inflammatory mediators should be distinguished from evidence of gasdermin-mediated pyroptotic cell death, as in other malignancies [216]. Computational analyses have also identified prognostic gene signatures associated with ferroptosis, pyroptosis, and cuproptosis, but these remain associative and require functional validation.

The role of autophagy is quite context-dependent. Although inhibition of autophagy may increase the susceptibility of cancer cells to treatment, basal autophagy can support tumor cell survival under nutrient deprivation or therapeutic stress [217]. Whether autophagy contributes to cell death or promotes survival depends on the experimental setting.

### 6.3. Cervical Cancer

Persistent high-risk HPV infection and expression of the viral oncoproteins E6 and E7 contribute to cervical carcinogenesis by disrupting cell-cycle regulation and apoptosis through pathways involving p53 and retinoblastoma protein (pRB) [218,219]. Consequently, resistance to apoptosis is an important feature of cervical cancer, providing a basis for the study of alternative forms of RCD.

An emerging body of evidence has investigated ferroptosis as a potential therapeutic strategy in cervical cancer. Experimental modulation of GPX4, SLC7A11, glutathione metabolism, and iron homeostasis can influence lipid peroxidation and cancer cell survival [220,221]. Some chemotherapeutic agents and targeted therapies have also been reported to increase susceptibility to ferroptosis, suggesting potential synergy with conventional anticancer treatments.

Pyroptosis may also have therapeutic potential because gasdermin activation can convert non-lytic cell death into inflammatory tumor-cell lysis. Caspase-mediated activation of gasdermins has been linked to increased responsiveness to chemotherapeutic drugs in certain cervical cancer cell lines [48,222]. However, the extent to which therapeutic effects result from direct tumor-cell killing, modulation of antitumor immunity, or both remains unclear.

Cuproptosis-related molecular signatures have also been reported in cervical cancer and associated with prognosis, immune infiltration, or drug response [223,224]. Nonetheless, these findings are predominantly computational, and direct evidence that copper-dependent aggregation of lipoylated mitochondrial proteins drives cell death in cervical tumors remains lacking.

### 6.4. Therapeutic Implications of RCD Targeting in Gynecological Cancer

A key therapeutic rationale for targeting RCD in gynecological malignancies is to exploit acquired cancer cell dependencies that arise during tumor adaptation and treatment resistance. Among non-apoptotic RCD modalities, ferroptosis is currently one of the most extensively investigated therapeutic targets [225,226]. Cancer cells with high metabolic activity and altered redox homeostasis may become particularly dependent on GPX4 activity, glutathione synthesis, or iron homeostasis for survival. Targeting ferroptosis in combination with other therapies may therefore help overcome treatment resistance and enhance tumor cell death [214].

Pyroptosis and necroptosis may offer an additional therapeutic advantage, as their lytic nature can promote the release of tumor antigens and danger signals, potentially enhancing antitumor immune responses. However, excessive or chronic inflammatory signaling may also exert pro-tumorigenic effects, indicating that therapeutic strategies should be considered within the context of the tumor immune microenvironment [206].

Cuproptosis is a promising but still nascent therapeutic concept. Its dependence on mitochondrial metabolism suggests that metabolically defined tumor-cell populations may exhibit differential susceptibility to copper-dependent cell death. However, therapeutic targeting of cuproptosis remains substantially less developed than ferroptosis-based approaches [227,228,229].

In summary, emerging RCD pathways provide therapeutic opportunities beyond conventional apoptosis-based strategies and may offer novel approaches to overcoming treatment resistance in gynecological cancers. However, most current evidence is derived from preclinical studies, particularly in vitro experiments and animal models, and clinically validated RCD-directed biomarkers and therapies remain limited. Future research should focus on establishing RCD pathway dependencies, identifying cancer-specific vulnerabilities, and determining the therapeutic efficacy of targeting specific RCD pathways.

## 7. Molecular Modulators and Environmental Determinants of RCD

The biological context in which cellular stress or damage occurs influences which RCD pathways are activated in reproductive cells. OS, mitochondrial dysfunction, inflammation, immune signaling, metabolic dysregulation, and epigenetic alterations are interconnected processes that may modulate the threshold for RCD activation [230]. Reproductive cells may be particularly susceptible to these interactions because gametogenesis, folliculogenesis, implantation, and placentation require substantial metabolic adaptation and tightly regulated redox and immune homeostasis. Environmental and lifestyle exposures may further modify these processes [231,232,233]. Therefore, identifying these shared mechanisms is essential for understanding why distinct reproductive disorders may involve similar RCD pathways.

### 7.1. OS and Redox Homeostasis

Redox regulation represents a key mechanistic link between reproductive physiology and RCD. ROS are involved in normal reproductive functions such as sperm capacitation, ovulation, embryo signaling, implantation, and placenta formation. The biological effects of ROS depend on their concentration, subcellular localization, duration of exposure, and the capacity of antioxidant systems to maintain or restore redox homeostasis [234,235]. OS occurs when ROS production exceeds antioxidant defenses, resulting in damage to lipids, proteins, DNA, and cellular organelles.

This balance is particularly relevant to ferroptosis, which, unlike nonspecific oxidative damage, is characterized by iron-dependent phospholipid peroxidation [38]. However, lipid peroxidation alone does not establish ferroptotic cell death, which requires evidence of involvement of ferroptosis-specific regulatory mechanisms [236].

OS can also promote RCD through additional signaling pathways. ROS can promote mitochondrial outer membrane permeabilization and apoptosis, enhance inflammasome activation, and modulate necroptotic signaling. Thus, OS associated with reproductive disorders may increase cellular susceptibility to multiple RCD pathways rather than selectively activating a single cell death modality [27,35].

### 7.2. Mitochondrial Dysfunction as a Hub of RCD Regulation

Mitochondria are involved in ATP generation, redox signaling, calcium homeostasis, biosynthetic processes, and cell death mechanisms. These functions are particularly important in reproduction. Oocyte maturation and early embryonic development depend on tightly regulated mitochondrial function, while fertilization requires adequate cellular energy metabolism, and trophoblast differentiation and placental development involve substantial metabolic adaptations [237].

The intrinsic apoptotic pathway is closely linked to mitochondrial function through BAX/BAK-mediated mitochondrial outer membrane permeabilization and the subsequent release of cytochrome c. However, mitochondrial dysfunction may influence not only apoptosis but also a broader network of RCD pathways [238]. Disruption of mitochondrial electron transport can increase ROS production and alter susceptibility to ferroptosis, while mitochondrial iron homeostasis and DHODH activity modulate ferroptotic sensitivity. Moreover, damaged mitochondria may generate signals that activate inflammatory processes [239].

Mitochondrial metabolism and cuproptosis have an even more direct association. Copper-dependent toxicity underlying cuproptosis involves lipoylated mitochondrial proteins of the tricarboxylic acid cycle, directly linking mitochondrial respiratory metabolism to susceptibility to this RCD pathway [210]. However, evidence regarding the physiological and pathophysiological relevance of cuproptosis in reproductive cells undergoing metabolic transitions remains limited.

Mitophagy represents one of the major regulatory processes through which defective mitochondria can be selectively removed [240]. Effective mitochondrial quality control may limit excessive ROS production and RCD activation, whereas impaired mitophagy may exacerbate OS and inflammatory signaling. Thus, mitochondrial dysfunction may act both upstream and downstream of RCD and should not be interpreted solely as a consequence of cell death [241].

### 7.3. Inflammation and Immune Signaling

Physiological inflammatory signaling is required during several reproductive processes, including ovulation, implantation, decidualization, and parturition. Reproductive success therefore depends on the appropriate regulation of inflammatory activity rather than its complete suppression. Chronic or excessive inflammatory signaling, on the other hand, may impair cell survival and promote inflammation-associated RCD [242,243,244].

Pyroptosis represents a direct mechanistic link between inflammation and RCD. Recognition of damage- and pathogen-associated molecular patterns can activate inflammasomes, leading to caspase activation, gasdermin cleavage, plasma membrane permeabilization, and the release of IL-1β, IL-18, and DAMPs. This bidirectional interaction may create a positive feedback loop in which inflammatory signaling promotes pyroptosis, while pyroptotic cell death further amplifies inflammation [48].

Necroptosis may also contribute to inflammation by inducing membrane damage and releasing DAMPs. Moreover, apoptosis, pyroptosis, and necroptosis are interconnected through shared regulatory molecules, including caspase-8 and RIP kinases. Under specific inflammatory conditions, these pathways may be coordinately activated, resulting in a PANoptotic response [245].

Such intercellular communication may be particularly important at the maternal–fetal interface, where immune tolerance must coexist with antimicrobial defense and tightly regulated inflammatory responses [246]. Decidual immune cells, trophoblasts, stromal cells, and endothelial cells engage in reciprocal communication, and dysregulation of RCD in one of these cell populations could influence the function of neighboring cells. However, detection of inflammatory cytokines or inflammasome activation alone is insufficient to establish the occurrence of inflammatory cell death [247,248].

Notably, the consequences of RCD depend on the cellular compartment in which it occurs. In reproductive cells, including germ cells, granulosa cells, endometrial cells, and trophoblasts, excessive RCD may directly impair gametogenesis, endometrial function, implantation, or placentation [2,73,144]. In contrast, RCD in resident or infiltrating immune cells may primarily modify the local inflammatory microenvironment through the release of cytokines, DAMPs, and other inflammatory mediators [19,203,245]. These processes can interact bidirectionally, as immune-cell-derived inflammatory signals may alter RCD susceptibility in reproductive cells, whereas injury or death of reproductive cells may further promote immune activation [2,246,248]. Therefore, tissue-level detection of RCD-associated markers does not identify the cellular compartment in which RCD occurs, an important limitation when interpreting findings from heterogeneous reproductive tissues.

### 7.4. Metabolic Regulation of Cell-Death Susceptibility

Cellular metabolism influences both resource availability and susceptibility to specific RCD pathways. Reproductive cells and tissues exhibit distinct metabolic profiles that change across developmental stages. Oocyte development depends on metabolic cooperation with surrounding granulosa and cumulus cells, while early embryonic development, decidualization, and trophoblast differentiation involve substantial shifts in substrate utilization, mitochondrial metabolism, and glycolysis [249,250,251].

Ferroptosis is closely linked to cellular metabolism. Amino acid metabolism influences cysteine availability for glutathione synthesis, lipid metabolism determines the availability of oxidizable phospholipids, and iron metabolism regulates the catalytic generation of ROS [252]. Glucose and mitochondrial metabolism can additionally affect antioxidant defenses and sensitivity to ferroptosis. Cuproptosis is also closely linked to cellular metabolism, as cells with greater dependence on mitochondrial respiration and lipoylated tricarboxylic acid cycle proteins exhibit increased susceptibility to this form of RCD [229].

Metabolic dysregulation may therefore indirectly influence RCD in reproductive cells and tissues. Conditions such as hyperglycemia, insulin resistance, obesity-associated dyslipidemia, and nutrient imbalances can alter mitochondrial function, ROS production, inflammatory signaling, and membrane lipid composition. These interactions provide a potential mechanism linking metabolic disturbances to reproductive cell injury, with different RCD pathways potentially predominating according to the cellular and disease context [24].

### 7.5. Genetic, Epigenetic, and Non-Coding RNA Regulation

Transcriptional and epigenetic mechanisms also contribute to the regulation of RCD. DNA methylation, histone modifications, chromatin accessibility, and non-coding RNAs may modulate the levels of cell death sensors, antioxidants, metabolic enzymes, and executioners. This regulation may be particularly relevant in reproductive tissues, where extensive epigenetic remodeling occurs during gametogenesis, embryonic development, and placentation [253].

Evidence indicates that miRNAs and lncRNAs regulate key molecular components of apoptosis, ferroptosis, pyroptosis, and autophagy. For instance, non-coding RNA networks can modulate the levels of GPX4, SLC7A11, NLRP3, BCL-2 family members, or autophagy-related proteins, thus affecting cellular stress resistance [254,255]. Epigenetic regulation of RCD-related genes may also contribute to persistent alterations in cellular phenotypes following environmental or metabolic exposures.

Nevertheless, regulation of RCD-related gene expression should be distinguished from direct regulation of the cell death phenotype itself. Changes in transcription, DNA methylation, or miRNA–target interactions may indicate molecular regulation but require functional validation to establish their effects on RCD execution [256]. This distinction is particularly important in multi-omics studies, in which RCD-related signatures are frequently identified without direct functional assessment of cell death.

### 7.6. Environmental and Lifestyle Exposures

Reproductive cells are particularly susceptible to environmental exposures that disrupt redox, mitochondrial, metabolic, endocrine, and inflammatory homeostasis. The effects of endocrine-disrupting chemicals (EDCs), heavy metals, air pollution, pesticides, plastic-associated chemicals, and other environmental toxicants have thus been studied as possible modulators of reproductive cell survival and injury [257].

Multiple environmental toxicants have been shown to increase ROS production, disrupt mitochondrial membrane potential and intracellular metal homeostasis, and impair antioxidant defense mechanisms. Depending on the exposure context, these alterations may promote apoptosis or increase cellular susceptibility to ferroptosis, pyroptosis, and other RCD pathways under conditions of cellular stress. Metal exposure warrants particular consideration because iron and copper homeostasis are closely linked to ferroptosis and cuproptosis, respectively. However, metal toxicity does not necessarily imply involvement of these pathways [258,259].

EDCs may also affect RCD via endocrine signaling. Disruption of estrogenic, androgenic, or metabolic signaling may impair folliculogenesis, spermatogenesis, endometrial function, and placental physiology by altering mitochondrial function, redox homeostasis, and RCD-related signaling. Experimental studies have investigated RCD mechanisms in reproductive toxicity induced by chemicals such as bisphenols, phthalates, and certain pesticides, although the concentrations used do not always reflect environmentally relevant exposure levels [260].

Lifestyle and metabolic factors may interact with these environmental exposures and converge on shared RCD-regulatory pathways. Diet, obesity, smoking, alcohol consumption, physical activity, and metabolic health can influence systemic OS and inflammation, thereby modifying the cellular environment in which RCD occurs. However, direct evidence linking these individual factors to the execution of specific RCD pathways in human reproductive tissues remains limited [261].

Overall, OS, mitochondrial dysfunction, inflammation, immune signaling, metabolic alterations, epigenetic regulation, and environmental exposures form an interconnected network that modulates RCD rather than acting as independent determinants. Interactions among these factors may influence both the extent of cell death and the specific RCD pathway involved. This framework may explain why similar molecular alterations are observed across distinct reproductive disorders despite differences in their initiating factors and clinical manifestations. In addition, this framework provides a biological rationale for therapeutic strategies targeting not only RCD execution machinery but also the cellular states that influence susceptibility to cell death [123].

## 8. Translational and Therapeutic Opportunities

Advances in understanding RCD in reproductive biology have highlighted opportunities for clinical translation through the development of RCD-related biomarkers and the therapeutic targeting of specific cell death pathways [124,135,171]. However, clinical translation requires a substantially higher level of evidence than the identification of pathway-associated molecular alterations. A therapeutic target should be supported by evidence that pathway modulation produces clinically meaningful effects with acceptable specificity, whereas a clinically useful biomarker should provide information beyond that captured by established diagnostic criteria. These considerations are particularly important in reproductive medicine because therapeutic interventions may affect gametes, embryos, the endometrium, or the placenta. Therefore, the translational value of RCD should be assessed according to the level of mechanistic and clinical validation rather than the number of pathway-associated genes or molecular signatures identified.

### 8.1. RCD-Associated Biomarkers and Multi-Omics Approaches

Molecular components of RCD pathways are increasingly being investigated as candidate biomarkers for reproductive disorders. Candidate markers include GPX4, SLC7A11, ACSL4, and iron-regulatory proteins for ferroptosis; NLRP3, caspase-1, and gasdermins for pyroptosis; RIPK3 and MLKL for necroptosis; and selected regulators of apoptosis and autophagy. These candidate biomarkers are being investigated across reproductive tissues and biological samples, including ovarian and endometrial tissues, granulosa cells, follicular fluid, semen and sperm, placental tissue, and peripheral blood [128,142,157,166,262].

The potential value of RCD-related biomarkers lies partly in their ability to provide biological information beyond that captured by conventional clinical parameters. Alterations in lipid and redox homeostasis may indicate cellular stress before functional tissue abnormalities become apparent, whereas inflammatory RCD-related biomarkers may reflect pathological processes at the maternal–fetal interface. However, several limitations currently restrict their clinical application. Studies vary considerably in sampling strategies and analytical methods; many candidate biomarkers have been evaluated only in small single-center cohorts, and disease-associated alterations may not be specific to a single RCD pathway. For example, increased NLRP3 expression may indicate inflammasome activation but does not establish pyroptosis, whereas reduced GPX4 activity reflects impaired antioxidant defense but does not confirm ferroptotic cell death [142,166,262].

From a clinical perspective, diagnostic performance remains insufficiently characterized for most proposed RCD-associated biomarkers in reproductive medicine. Sensitivity, specificity, clinically validated cut-off values, and external validation are generally unavailable, and most human studies remain exploratory or associative. Some multigene signatures have shown promising discriminatory performance; for example, a ferroptosis-related transcriptomic model in RIF achieved an area under the receiver operating characteristic curve of 0.891 [171]. However, such models require independent validation in larger and clinically well-characterized populations before their diagnostic utility can be established. Therefore, current RCD-associated biomarkers should primarily be regarded as candidate molecular or stratification markers rather than established diagnostic tests.

Multi-omics approaches extend beyond individual biomarkers by integrating data from transcriptomics, proteomics, metabolomics, lipidomics, and epigenomics. These approaches may be particularly valuable for studying RCD because cellular susceptibility to death is determined by interactions among metabolic and signaling pathways rather than by individual molecules. For instance, ferroptosis is regulated by iron, phospholipid, and amino acid metabolism together with cellular antioxidant defenses, whereas cuproptosis is closely linked to mitochondrial metabolism and protein lipoylation. Hence, integrated molecular profiling may identify RCD-associated phenotypes that cannot be detected by gene or protein analysis alone [157].

Single-cell and spatial approaches may provide additional insights into RCD mechanisms within heterogeneous reproductive tissues. Bulk analysis of the endometrium, ovary, testis, or placenta may obscure cell type-specific differences among epithelial, stromal, immune, vascular, germ, and trophoblast populations. Single-cell RNA sequencing and spatial profiling may identify the cellular compartments enriched in RCD-associated molecular programs and characterize their relationships with immune and stromal cell populations. Such approaches are particularly valuable when opposing survival and death programs coexist within the same tissue [263,264,265].

Multi-omics approaches also present important methodological challenges. Batch effects arising from differences in sample processing, analytical platforms, and sequencing protocols can introduce technical variation that may be difficult to distinguish from biological differences. Tissue heterogeneity represents an additional concern, particularly in complex reproductive tissues containing multiple cell populations with distinct molecular profiles [263,264,265]. Moreover, high-dimensional datasets combined with relatively small sample sizes increase the risk of computational overfitting and may generate signatures that perform well in discovery cohorts but lack reproducibility. Rigorous normalization, appropriate computational validation, and, importantly, independent validation in external and clinically well-characterized cohorts are therefore essential before multi-omics-derived RCD signatures can be considered reliable or clinically applicable [256].

However, computational identification of an RCD-associated molecular signature should be regarded as a discovery and stratification tool rather than evidence that the corresponding cell-death program is functionally active. Predictive models based on ferroptosis-, cuproptosis-, or PANoptosis-related gene signatures may have potential clinical utility even without demonstrating activation of the corresponding RCD pathway. Accordingly, predictive performance should be distinguished from mechanistic evidence of pathway execution and dependency [171].

### 8.2. Therapeutic Modulation of RCD Pathways

Therapeutic modulation of RCD follows two opposing principles: inhibition of inappropriate cell death to preserve reproductive function in non-malignant disorders and induction of RCD to eliminate tumor cells in gynecological malignancies. Among emerging therapeutic targets, ferroptosis is currently one of the most extensively investigated RCD pathways in reproductive medicine [3,101,146,158]. Experimental ferroptosis inhibitors, iron chelators, and interventions targeting the glutathione–GPX4 axis, have demonstrated protective effects in models of ovarian, testicular, and placental injury [133,150,153,266]. However, many proposed therapies exert antioxidant, anti-inflammatory, or metabolic effects; therefore, changes in OS biomarkers alone do not establish that their therapeutic effects are mediated through ferroptosis [1,91]. Importantly, therapeutic responsiveness to modulation of an RCD pathway does not by itself establish that the pathway is a primary causal driver of the underlying disease.

The therapeutic potential of other RCD pathways in reproductive medicine remains largely unexplored. Inhibition of inflammasome–caspase–gasdermin signaling may reduce pyroptosis-associated inflammatory injury, whereas targeting RIPK1/RIPK3/MLKL signaling may protect against necroptosis-mediated tissue damage [145,184,185,267]. Particular caution is required when considering autophagy as a therapeutic target because it frequently functions as an adaptive and cellular quality-control mechanism rather than as an execution pathway for cell death [62,63,65].

In gynecologic cancer, the aim of treatment is the opposite. Induction of ferroptosis may provide a strategy to overcome apoptosis resistance [199,200,201,225], whereas activation of pyroptosis or necroptosis may combine tumor cell elimination with enhanced antitumor immune responses [206,207,226]. The therapeutic exploitation of cuproptosis in metabolically susceptible tumors is also under investigation [57,58,227,228,229]. However, these approaches remain predominantly experimental, and selective targeting of malignant cells while limiting injury to normal tissues will be critical [194,195,225,226]. Representative primary intervention studies evaluating therapeutic modulation of RCD pathways in reproductive and gynecological disorders are summarized in Table 3, with emphasis on the experimental model, mechanistic controls, and strength of evidence.

Translation of RCD-targeted therapies also requires careful consideration of their spatial and temporal specificity. Systemic modulation may affect not only the pathological cell population but also gametes, granulosa cells, endometrial cells, trophoblasts, immune cells, and embryonic or fetal tissues [2,70,71,72,73,74,75,76,77,78,79,80,81,82,83,84,85,86,93,94,95,96,97,98,99,100,101]. The consequences may further depend on treatment timing relative to the reproductive cycle, implantation, and gestation, particularly because physiological RCD contributes to tissue remodeling, immune regulation, and developmental cell turnover [2,81,82,83,84,85,86,87,88,89,90,91,92,93,94,95,96,99,100,101,102,103,104,105]. Maternal and embryonic/fetal exposure should therefore be evaluated separately. Local rather than systemic delivery, limited treatment duration, reversibility, and rigorous assessment of reproductive and developmental toxicity may be necessary to minimize disruption of physiological RCD while retaining therapeutic efficacy.

Overall, a substantial translational gap remains between experimental RCD modulation and its clinical application in the treatment of reproductive disorders. Most evidence is derived from cell culture and animal models, while pharmacological interventions often lack specificity for individual RCD pathways. Functional rescue and genetic manipulation provide stronger evidence of RCD pathway dependency than changes in pathway-associated markers [1,26], while reproductive efficacy and safety ultimately require validation in human studies.

### 8.3. Toward RCD-Informed Precision Reproductive Medicine

Given the biological heterogeneity of reproductive disorders, targeting a single RCD pathway may be insufficient to achieve therapeutic benefit. Moreover, patients with the same reproductive disorder may exhibit substantial biological heterogeneity in redox homeostasis, inflammatory signaling, metabolism, and susceptibility to cell death. Identifying molecular subgroups characterized by dysregulation of specific RCD pathways may therefore provide a more appropriate strategy for patient stratification [15,24,256].

Such stratification requires more than the detection of RCD-associated markers. Evidence of pathway activation should be supported by identification of the underlying mechanism and functional demonstration that modulation of the specific pathway alters the reproductive phenotype [1,26]. Combining functional assays with multi-omics profiling may help identify predominant redox, inflammatory, metabolic, or mitochondrial phenotypes and determine their association with specific RCD pathways [256,263,264,265].

Cell type and temporal specificity are also critical, as RCD pathways may exert distinct physiological or pathological effects across reproductive stages and cellular compartments [2,263,264,265]. Therapeutic strategies should therefore target validated RCD pathways within the appropriate biological context and temporal window rather than broadly inhibiting or inducing cell death.

Ultimately, the potential of RCD-informed precision reproductive medicine depends on identifying when, where, and in which patients a specific cell-death pathway represents a causal and therapeutically actionable component of reproductive disease. Although this framework remains largely conceptual, validation of distinct RCD-associated phenotypes may provide a basis for more precise patient stratification and the development of targeted diagnostic and therapeutic strategies.

## 9. Future Perspectives

Despite the growing evidence linking RCD to reproductive disorders, the strength of evidence varies considerably across RCD modalities and reproductive conditions, and substantial methodological and translational challenges remain. A major limitation of the current literature is that mechanistic involvement is frequently inferred from RCD-associated molecular markers or transcriptional signatures rather than demonstrated through functional evidence of pathway execution. A specific RCD phenotype cannot be established solely on the basis of altered expression of GPX4, SLC7A11, NLRP3, RIPK3, MLKL, or other RCD-associated proteins. Future studies should therefore provide evidence across multiple levels and, where feasible, demonstrate pathway dependency through functional rescue, pharmacological inhibition, or genetic manipulation of the specific RCD machinery [1,91,256].

Increased attention should be paid to human-based, cell type-specific, and temporally resolved data. Reproductive tissues comprise heterogeneous cell populations that may exhibit distinct or even opposing responses to RCD-related stimuli [263,264,265]. Moreover, the biological consequences of RCD may differ across reproductive stages, with a pathway contributing to physiological remodeling in one context but pathological cell loss in another. This issue is particularly important in RIF, RPL, and placental disorders, because samples obtained after implantation failure, pregnancy loss, or disease onset cannot establish whether observed RCD alterations are causal events or secondary consequences of tissue injury.

Better experimental models will play an important role in overcoming these limitations. Prospective and longitudinal human studies, together with organoids, ex vivo systems, and other physiologically relevant models, may better capture cellular interactions in human reproduction than chemically induced injury models [94,263]. Such approaches may facilitate the identification of RCD-associated molecular phenotypes while simultaneously enabling assessment of their functional relevance.

Therapeutic research must focus on specificity, reproductive safety, and clinically relevant outcomes. Current evidence for RCD-targeted interventions comes largely from preclinical studies, with ferroptosis receiving the greatest attention [101,158,179]. Many candidate modulators also have antioxidant, anti-inflammatory, or metabolic effects that are not specific to RCD. Pathway-specific genetic manipulation and functional rescue experiments will therefore be important for establishing causality and therapeutic mechanism. Ultimately, clinical translation will require prospective studies evaluating clinically relevant outcomes, including oocyte competence, embryo development, implantation, pregnancy loss, live birth, semen parameters, and maternal and fetal outcomes, rather than relying solely on surrogate measures.

A critical future objective will be to determine whether functionally validated RCD phenotypes can support biologically informed patient stratification. Integrating functional RCD assessment with clinical characteristics and multi-omics profiles may eventually enable the identification of patients in whom a specific RCD pathway contributes to reproductive dysfunction and represents a viable therapeutic target.

## 10. Conclusions

RCD is an integral component of reproductive homeostasis, and its dysregulation contributes to reproductive pathology. Apoptosis remains the most extensively studied RCD pathway in both reproductive physiology and pathology. In contrast, autophagy primarily contributes to cellular adaptation, homeostasis, and quality control rather than functioning as a cell death mechanism. Among the emerging RCD modalities, ferroptosis has accumulated substantial evidence across ovarian disorders, male reproductive dysfunction, implantation failure, pregnancy loss, and placental disorders, whereas pyroptosis has been primarily implicated in inflammation-associated reproductive pathology.

The relevance of these pathways reflects their close interaction with biological processes central to reproduction, including redox homeostasis, mitochondrial function, inflammation, immune signaling, and metabolism. The effects of these pathways are highly context-dependent, with RCD contributing to physiological remodeling, pathological cell loss, or, particularly in cancer, a cellular vulnerability that can potentially be therapeutically exploited. Therefore, the identification of RCD-associated biomarkers does not establish execution of a specific cell death program, and mechanistic evidence should be clearly distinguished from computational associations.

RCD-related biomarkers and therapeutic strategies show considerable potential, but clinical translation remains limited by insufficient functional validation and the predominance of preclinical evidence. Future studies should prioritize functional validation of RCD pathways in human reproductive tissues, establish their causal contribution to disease, and determine whether pathway-specific modulation can safely improve clinically relevant reproductive outcomes. Ultimately, a more precise understanding of RCD may identify therapeutically actionable cellular vulnerabilities and support biologically informed approaches to the diagnosis and treatment of reproductive disorders.

## Figures and Tables

**Table 1:** Molecular indicators and experimental approaches for the identification and functional validation of major regulated cell death pathways.

RCD Pathway	Common Molecular/Biochemical Indicators	Confirmatory Approaches	Functional Validation
Apoptosis	Cleaved caspase-3/7, cleaved PARP, BAX/BAK activation, cytochrome *c* release	Caspase activity; Annexin V; TUNEL/DNA fragmentation; characteristic apoptotic morphology	Rescue by caspase inhibition; genetic manipulation of core apoptotic machinery (e.g., BAX/BAK or caspases)
Ferroptosis	GPX4↓, SLC7A11↓, ACSL4↑, GSH depletion, iron accumulation, lipid peroxidation	Lipid ROS/peroxidation assays; labile iron assessment; characteristic biochemical/morphological changes	Rescue by ferroptosis inhibitors (e.g., ferrostatin-1/liproxstatin-1) or iron chelation; genetic manipulation of GPX4, SLC7A11, ACSL4, or related machinery
Pyroptosis	Inflammasome activation, cleaved caspase-1, GSDMD cleavage, IL-1β/IL-18 maturation/release	Detection of gasdermin cleavage/pore formation; membrane permeabilization; cytokine release	Inhibition or genetic disruption of inflammasome–caspase–gasdermin machinery with rescue of cell death
Necroptosis	RIPK1/RIPK3 activation, phosphorylated RIPK3, phosphorylated MLKL	MLKL phosphorylation and membrane translocation/oligomerization; membrane-disruptive morphology	Rescue by pathway-specific inhibition; genetic disruption of RIPK3 or MLKL
Autophagy-dependent cell death	LC3-II accumulation, p62/SQSTM1 changes, ATG protein activity, increased autophagic structures	Assessment of autophagic flux rather than static markers alone; lysosomal/autophagosome assays	Demonstration that inhibition or genetic disruption of autophagy machinery prevents cell death
Cuproptosis	Copper accumulation, lipoylated TCA-cycle protein aggregation, loss of Fe–S proteins, FDX1-related changes	Assessment of copper dependence and lipoylated protein aggregation/proteotoxic stress	Rescue by copper chelation or genetic manipulation of cuproptosis-related machinery (e.g., FDX1/lipoylation pathway)
PANoptosis	Concurrent activation of apoptotic, pyroptotic, and necroptotic machinery; PANoptosome components	Demonstration of coordinated activation of multiple RCD execution pathways in the same biological context	Combined genetic/pharmacological approaches demonstrating functional interdependence of pyroptotic, apoptotic, and necroptotic machinery

**Table 3:** Representative primary intervention studies targeting RCD pathways in reproductive and gynecological disorders.

Author, Year	Condition	Study Design/Model	RCD Pathway	Intervention/Target	Comparator/ Mechanistic Control	Main Findings	Evidence Type/Strength
Li et al., 2021 [192]	Endometriosis	Primary human stromal cells; pharmacological/genetic experiments; mouse endometriosis model	Ferroptosis	Erastin; FPN modulation	DFO, Fer-1, liproxstatin-1; FPN overexpression/knockdown	Ferroptosis inhibitors attenuated erastin cytotoxicity; FPN manipulation bidirectionally altered ferroptosis. Erastin induced regression of ectopic lesions in mice	Primary human cells + animal model|Functionally validated: ferroptosis-specific pharmacological rescue and FPN genetic manipulation, with in vivo lesion regression
Li et al., 2024 [134]	PCOS	Primary human GCs; DHT-treated GCs; DHEA-induced PCOS rats	Ferroptosis	Fer-1; RSL3; NCOA4-dependent ferritinophagy	Fer-1; RSL3; siRNA manipulation	DHT promoted ferroptosis through NCOA4-dependent ferritinophagy. Fer-1 improved metabolic, hormonal and ovarian PCOS features, whereas RSL3 induced PCOS-like abnormalities	Primary human granulosa cells + animal model|Functionally validated: Fer-1 rescue and RSL3 induction support ferroptosis dependency, with NCOA4 knockdown supporting the ferritinophagy mechanism
Du et al., 2023 [152]	Cisplatin-induced POI	Cisplatin-induced POI rats and ovarian GC experiments	Ferroptosis-associated ovarian injury	Vitamin E	Injury versus vitamin E; no pathway-specific rescue/genetic control	Vitamin E attenuated ferroptosis-associated alterations and improved follicular development, fibrosis, hormone profiles and reproductive function	Animal model + granulosa cell experiments|Mechanistically supportive: reproductive rescue with attenuation of ferroptosis-associated changes, but without ferroptosis-specific rescue or genetic validation
Niu et al., 2023 [266]	Oxidative ovarian injury	3-NP mouse model; H_2_O_2_-exposed GCs; porcine GC RNA-seq	Ferroptosis-associated ovarian injury/apoptosis	Spermidine; Nrf2/HO-1/GPX4 and Akt/FHC/ACSL4	Injury/dose controls; no ferroptosis-specific rescue	Spermidine improved ovarian reserve and reproductive function and attenuated ferroptosis-associated molecular changes	Animal model + granulosa cell models|Mechanistically supportive: reproductive rescue with attenuation of ferroptosis-associated changes, but without ferroptosis-specific functional rescue or genetic validation
Hu et al., 2025 [127]	Ovarian aging/DOR; CTX-induced ovarian failure	Human GCs; GC experiments; aged and CTX-treated mice	Ferroptosis	Fer-1; GPX4-associated ferroptosis	Ferroptosis inducers/inhibitors	Fer-1 improved oocyte yield/quality in aged mice and partially rescued follicular reserve and oocyte outcomes after CTX	Patient-derived granulosa cells + cell model + animal models|Functionally validated: pharmacological ferroptosis modulation with Fer-1-mediated rescue of granulosa-cell dysfunction and ovarian/reproductive phenotypes
Chen et al., 2025 [153]	Chemotherapy-induced POI	CTX-induced POI rats; theca interna cells; exosomal miR-26a-5p manipulation	Ferroptosis-associated ovarian injury	hUCMSC-Exo; miR-26a-5p/PTEN/GPX4	miR-26a-5p-inhibited exosomes	Exosomes improved ovarian structure/function and reduced ferroptosis-associated changes; miR-26a-5p inhibition impaired their effect	Animal model + primary theca interna cells|Mechanistically supportive: miR-26a-5p manipulation supports the PTEN/GPX4 mechanism and ovarian rescue, but ferroptosis-specific pathway dependency was not directly demonstrated
Wu et al., 2025 [133]	Cisplatin-induced POF	Rat model and GC experiments	Ferroptosis	Phoenixin-14; ATF4/SLC7A11/GPX4	Fer-1; PNX-14 + Fer-1; ATF4 silencing	PNX-14 improved ovarian injury; ATF4 silencing reversed PNX-14-mediated improvements in GC viability and ferroptosis	Granulosa cell model + animal model|Functionally validated: Fer-1 comparator and ATF4 silencing support ferroptosis-dependent protection through the ATF4/SLC7A11/GPX4 axis
Li et al., 2026 [174]	RPL	RPL mouse model; erastin-exposed trophoblasts	Ferroptosis	Baicalein; Nrf2/GPX4	Fer-1, DFO, RSL3; Nrf2 silencing	Baicalein reduced fetal loss and placental damage; GPX4 inhibition diminished protection and Nrf2 silencing abolished its anti-ferroptotic effect	Trophoblast cell model + animal model|Functionally validated: ferroptosis-specific pharmacological controls and Nrf2/GPX4 pathway disruption, with in vivo pregnancy rescue
Zhu et al., 2025 [175]	RPL/early pregnancy loss	Human RPL placentas; primary trophoblasts; BeWo cells; LPS mouse model	Ferroptosis	Lactate; MCT1/PI3K–AKT–mTOR/SCD1/GPX4	Modulation of lactate transport/signaling	Lactate reduced STB ferroptosis susceptibility and rescued placental ferroptosis and fetal loss in mice	Patient placental tissue + primary human trophoblasts + trophoblast cell model + animal model|Functionally validated: modulation of ferroptosis susceptibility through the lactate–MCT1 signaling axis, with in vivo rescue of placental ferroptosis and pregnancy loss
Yang et al., 2022 [124]	Preeclampsia	Human PE placenta; FTL-knockdown pregnant rats; trophoblasts/explants	Ferroptosis	Fer-1; FTL-associated iron homeostasis	FTL knockdown; Fer-1 rescue	FTL knockdown induced ferroptosis, impaired spiral artery remodeling and PE-like phenotypes; Fer-1 significantly rescued these abnormalities	Patient placental tissue + trophoblast/explant models + animal model|Functionally validated: FTL deficiency induced ferroptosis and PE-like pathology, with Fer-1-mediated rescue of ferroptosis and impaired spiral artery remodeling
Yang et al., 2026 [180]	Preeclampsia	Clinical PE data; trophoblasts; PE-derived organoids; RUPP mice	Ferroptosis with concomitant apoptosis	DFO, MitoQ, Fer-1	Fer-1; Z-VAD	DFO and MitoQ rescued iron-overload injury in trophoblasts and PE-derived organoids; MitoQ reduced placental lipid-peroxidation markers in vivo	Patient-derived data + trophoblast cell model + placental organoid + animal model|Mechanistically supportive: pharmacological rescue across human-derived and experimental models, but limited demonstration of ferroptosis-specific in vivo pathway dependency
Zhao et al., 2026 [150]	Radiation-induced ovarian injury	Abdominal-irradiation rat model	Ferroptosis	S1P; Nrf2-GCLC-GPX4	BSO-mediated GCLC inhibition; Fer-1	S1P preserved ovarian structure, follicles and hormone profiles and reduced ferroptotic injury; BSO abolished protection	Animal model|Functionally validated: Fer-1 rescue and GCLC inhibition support ferroptosis dependency through the Nrf2–GCLC–GPX4 axis, with preservation of ovarian structure and function
Liu et al., 2024 [154]	CTX-induced POI	CTX-induced POI rats; LPS/nigericin-treated KGN cells	Pyroptosis	α-Ketoglutarate; NLRP3	MCC950; LPS/nigericin	AKG improved ovarian reserve and reduced NLRP3, GSDMD, caspase-1, IL-18 and IL-1β; MCC950 produced similar effects in vitro	Granulosa cell model + animal model|Mechanistically supportive: NLRP3 inhibition reproduced the protective effects on pyroptosis-associated changes, but direct genetic or gasdermin-specific demonstration of pyroptotic pathway dependency was not provided
Lin et al., 2025 [267]	Preeclampsia	Human PE placentas; LPS-induced PE mice; HTR-8/SVneo cells	Pyroptosis	Resveratrol; SIRT1/NLRP3–caspase-1–GSDMD	PE/LPS controls; no pathway-specific loss-of-function reported	Resveratrol improved PE and pregnancy outcomes while increasing SIRT1 and reducing NLRP3, caspase-1, GSDMD, ASC and inflammatory cytokines	Patient placental tissue + trophoblast cell model + animal model|Mechanistically supportive: in vivo reproductive rescue with suppression of the SIRT1/NLRP3–caspase-1–GSDMD axis, but without pathway-specific loss-of-function validation
Huang et al., 2026 [262]	Early-onset preeclampsia	Human EOPE samples; trophoblast cell lines; placental organoid/macrophage models; WT and Gsdme−/− pregnant mice	CASP3/GSDME-mediated pyroptosis	Vitamin D; GSDME-associated pyroptosis	Gsdme−/− versus WT mice; mechanistic trophoblast/organoid models	CASP3/GSDME-mediated trophoblast pyroptosis promoted inflammatory macrophage polarization and systemic inflammation; the latter occurred in WT but not Gsdme−/− mice. Vitamin D suppressed GSDME activation and trophoblast pyroptosis	Patient samples + trophoblast cell models + placental organoid/macrophage models + genetic animal model|Functionally validated: CASP3/GSDME-mediated pyroptosis demonstrated by genetic Gsdme deletion, with mechanistic validation across experimental models
Hu et al., 2024 [129]	Preeclampsia	Human PE placentas; HTR-8/SVneo trophoblast experiments; in vivo PE model	Necroptosis	THBS1; NEDD4-mediated TAK1 ubiquitination	Nec-1 and GSK’872 necroptosis inhibitors; Z-VAD-FMK pan-caspase inhibitor; THBS1 manipulation	THBS1 silencing induced trophoblast necroptosis and DAMP release and impaired trophoblast functions. Nec-1 and GSK’872 restored trophoblast survival, whereas Z-VAD-FMK did not. THBS1 regulated TAK1 stability through NEDD4-mediated K48-linked ubiquitination and attenuated PE phenotypes and placental necroptosis in vivo	Patient placental tissue + trophoblast cell model + animal model|Functionally validated: pathway-specific RIPK1/RIPK3 inhibition rescued THBS1-loss-induced necroptosis, with apoptosis-discriminating control, defined NEDD4–TAK1 mechanism, and in vivo disease rescue

## Data Availability

No new data were created or analyzed in this study. Data sharing is not applicable to this article.
